# Application of Quantum Chemical Topology Force Field FFLUX to Condensed Matter Simulations: Liquid Water

**DOI:** 10.1021/acs.jctc.2c00311

**Published:** 2022-08-08

**Authors:** Benjamin C. B. Symons, Paul L. A. Popelier

**Affiliations:** Department of Chemistry, University of Manchester, Oxford Road, Manchester M13 9PL, Great Britain

## Abstract

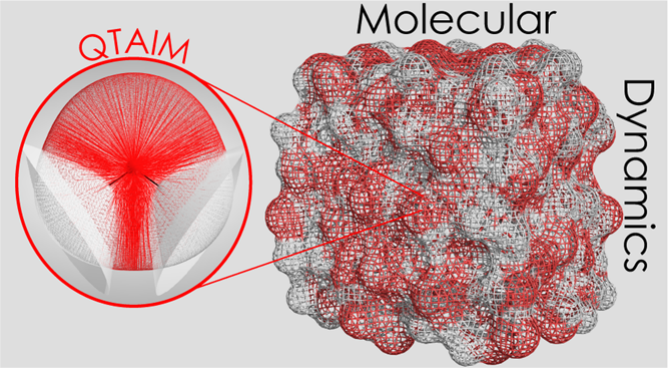

We present here the first application of the quantum chemical topology force field FFLUX to condensed matter simulations. FFLUX offers many-body potential energy surfaces learnt exclusively from *ab initio* data using Gaussian process regression. FFLUX also includes high-rank, polarizable multipole moments (up to quadrupole moments in this work) that are learnt from the same *ab initio* calculations as the potential energy surfaces. Many-body effects (where a body is an atom) and polarization are captured by the machine learning models. The choice to use machine learning in this way allows the force field’s representation of reality to be improved (*e.g*., by including higher order many-body effects) with little detriment to the computational scaling of the code. In this manner, FFLUX is inherently future-proof. The “plug and play” nature of the machine learning models also ensures that FFLUX can be applied to any system of interest, not just liquid water. In this work we study liquid water across a range of temperatures and compare the predicted bulk properties to experiment as well as other state-of-the-art force fields AMOEBA(+CF), HIPPO, MB-Pol and SIBFA21. We find that FFLUX finds a place amongst these.

## 1 Introduction

The best performing modern force fields are typically characterized by several important features. Firstly, they tend to allow molecules to be flexible rather than rigid. Secondly, it is increasingly the case that potential energy surfaces (PES) are fitted using at least some *ab initio* data. Some force fields such as AMOEBA+^1^ blend *ab initio* and experimental data when fitting whereas others use only *ab initio* data. For example, the two- and three-body terms of the MB-Pol potential are fitted using exclusively CCSD(T) data.^2,3^ Thirdly, it is now relatively common to include multipole moments thereby abandoning the paradigm of point charges. For example, the AMOEBA+, SIBFA21^4^ and HIPPO^5^ force fields have permanent atomic multipole moments up to and including quadrupole moments. Fourthly, there is also a considerable emphasis on making at least some of the multipole moments polarizable. Polarization is achieved in several different ways depending on the force field. The aforementioned AMOEBA+, SIBFA21 and HIPPO potentials have induced dipole moments while the MB-Pol potential also includes explicit three-body polarization.^6^ The recent AMOEBA+(CF) potential^7^ extends the AMOEBA+ potential to include so-called “charge flux”, *i.e*., charges that change with molecular geometry. Finally, there has been a push towards capturing many-body effects. All of the aforementioned force fields make at least some effort to include many-body effects. SIBFA21 and AMOEBA+ capture many-body effects with their induced dipole moments as well as charge transfer terms. However, it is MB-Pol that captures many-body effects in the most comprehensive manner, with considerable success.^8^ Note that there are many other successful modern force fields that have not yet been mentioned such as MB-UCB^9^ and the TTM family of force fields^10^ among others.^11,12^

To summarize, in order for a force field to be considered state-of-the-art it should be grounded in quantum mechanics, be flexible, and include many-body effects and high-rank polarizable multipole moments. The novel force field FFLUX is well positioned to contend in this space as it has all of these attributes. Alongside the essential features of a modern force field, FFLUX offers three more features that are key to its current and future success. Firstly, the parameterization of the force field for different systems is not tied to the functional form of the potential. Instead, the problem of parameterization is exported to a machine learning (ML) problem. The architecture of FFLUX is such that machine learning models can be used in a “plug and play” fashion. This means that an ML model can be made for any molecule of interest and plugged into FFLUX without requiring any modification of FFLUX itself. In this way, FFLUX approaches the ideal of a universal force field. Secondly, the level of approximation in the force field (*e.g*., the extent of the polarization and many-body effects) is tied into the ML models. This means that the approximations are well understood and controlled and that the level of approximation can be reduced in a systematic manner. Moreover, these improvements can be made without worsening the computational scaling of FFLUX thus offering a future-proof strategy, which will be explained in full in the Methods section. Finally, FFLUX is unique in that it utilizes the parameter-free atoms of quantum chemical topology (QCT).^13,14^ QCT is particularly well suited for a force field that operates at the atomic level because the theory establishes a properly defined atomic kinetic energy. Secondly, all atomic properties (charge, dipole, kinetic energy, potential energies) come from a single overarching three-dimensional (3D) integral over an atomic volume (or a six-dimensional (6D) integral for interatomic potential energies). This is important in the case of the multipole moments as it means that they are derived from the same *ab initio* calculations as the PESs. There is no need to introduce an additional, separate scheme (*e.g*., Hirshfeld^15^ or iterative stockholder atoms^16^) for multipole moments that is generally not rooted in quantum mechanics.

This paper represents the first foray of the FFLUX force field into bulk simulations. However, the fundamental components of the methodology have been developed and validated from the bottom up over the course of many years.^17−21^ At last, FFLUX brings everything together and the entire construction is validated against the most important arbiter of success: experimental data. The initial test case of water is an obvious choice. Water is an important solvent for biological systems and so it is of genuine utility to have accurate water models that can be combined with simulations of more complex molecules. Furthermore, water is very well studied experimentally and so there is a wealth of available data to validate against.

## 2 Methods

The key elements of the FFLUX methodology have been explained elsewhere.^19,20,22^ We will give a brief but comprehensive overview of the important details in this section. As with most force fields, the interactions in FFLUX can be divided into short-range and long-range. Note that the FFLUX force field is currently implemented in the DL_FFLUX code, which is a combination of DL_POLY 4 and the FFLUX force field.

### 2.1 Short-Range Interactions

**Figure 1:**
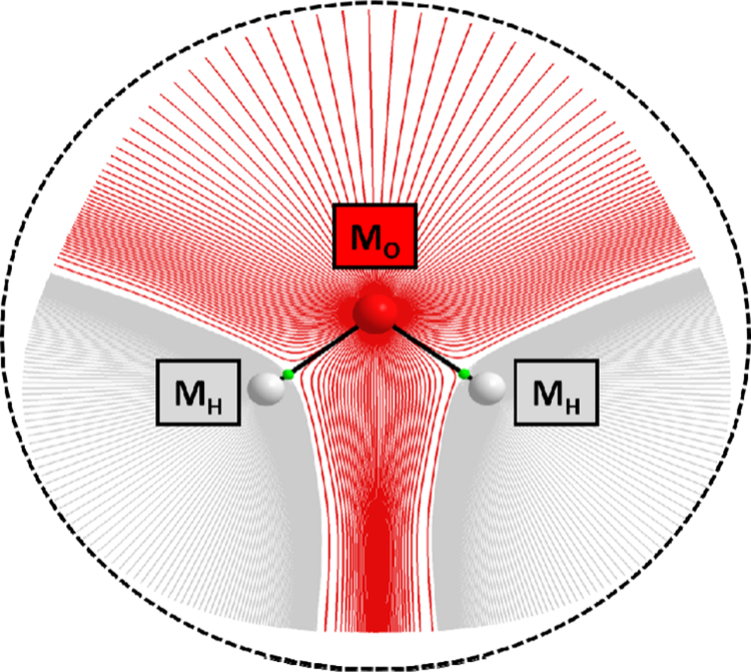
Diagram of a single water molecule in FFLUX. M_O_ and M_H_ represent machine learning models. The gradient paths of the electron density are shown for each atom. The dashed black line shows the boundary of the machine learning models (see main text) and should not be confused with a circle marking a cut-off radius. Gradient paths were visualized using AIMSTUDIO.^24^

The short-range interactions in FFLUX are handled by machine learning models, that is, Gaussian process regression (GPR) models. Figure 1 shows a representation of a single water molecule in FFLUX. Each atom is endowed with a number of GPR models, each of which predicts an atomic property such as atomic energies and atomic (point-)multipole moments. These models are denoted M_O_ and M_H_ for oxygen and hydrogen atoms, respectively. The GPR models learn atomic properties that are the output of quantum chemical topology calculations. The gradient paths that map out the QCT atomic basins are shown in Figure 1. The atomic energies are obtained using the interacting quantum atoms (IQA) energy partitioning scheme,^23^ which falls under the arch of QCT.

A given GPR model predicts properties for a single atom but it has “knowledge” of its surroundings. In the case of the water molecule shown in Figure 1, the boundary of the system that informs the predictions made by the models is shown by the dashed black line. In other words, the predictions made by M_O_ depend not only on the oxygen but also the two hydrogen atoms. In general, the prediction of a property Q_i_ belonging to atom *i*, by a model *M*_*i*_^Q^, will depend on some collection of *N*_atom_ atoms that make up the local environment. Note that the model now has a superscript *Q* because each property has its own model, *i.e*., each atom has a set of models sitting on it, one for each property that is predicted. The position vectors **r** of each of the *N*_atom_ atoms that comprise the local environment are converted into a feature vector **f**, where each feature *f*_*j*_ is a function of some subset of the atomic positions. For example, feature 1 is the distance between atoms 1 and 2, and as atom 3 is not involved, this feature is a function of the subset{1, 2}. The feature vector is *N*_feat_ = 3*N*_atom_ – 6 dimensional and serves as input to the GPR models as demonstrated in eq 1,
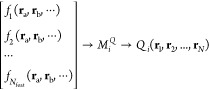
1

The full details of the feature definitions are given elsewhere.^19^ In this scheme the predicted, short-range energy for a given atom is a combination of the intra-atomic energy as well as the energy of the interaction between the atom and the *N*_atom_ – 1 atoms that fall within the bounds of its model (*i.e*., the other atoms that fall within the black boundary of Figure 1). The energy can be written more explicitly as
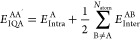
2

The IQA energy,*E*_IQA_^AA′^, for atom A has the superscript AA′ because it comprises the intra-atomic energy, *E*_Intra_^A^, as well as the interaction between atom A and all other atoms within the model boundaries labeled as A′. This second term is expressed in eq 2 as the sum of the interatomic interaction energies, *E*_Inter_^AB^. The intra-atomic energy, *E*_Intra_^A^, and the inter-atomic energies, *E*_Inter_^AB^, provide chemical insight but, for the purposes of the FFLUX force field, only *E*_IQA_^AA′^ is needed. The result of eqs 1 and 2 is that there is a predicted quantum mechanical, short-range PES that is inherently many-body in nature. Note that here a body stands for an atom and more details can be found in reference.^22^ This PES can be analytically differentiated with respect to the coordinates of each of the atoms in the short-range environment leading to short-range forces. Unlike more traditional force fields, in FFLUX there is no need for harmonic bond or angle potentials and other terms because the full intra-molecular description is provided by the predicted PES and its derivatives, giving rise to many-body intra-molecular energies and forces.

### 2.2 Long-Range Interactions

The long-range potential in FFLUX is split into an electrostatic and a van der Waals term, as shown in eq 3. The point at which the transition from short-range to long-range interactions occurs is determined by the boundary of a GPR model. For the case of so-called monomeric modeling (this is the case shown in Figure 1), the boundary of a model is synonymous with the boundary of a molecule. This means that all intra-molecular interactions are considered short-range and handled by the GPR models. All of the inter-molecular interactions are then considered long-range and handled according to eq 3,
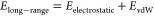
3

Monomeric modeling is employed in this paper but it is possible to extend to dimeric modeling or *N*-meric modeling in general where *N* is the number of molecules. In the case of *N*-meric modeling, the intermolecular interactions between a molecule and its nearest *N* – 1 neighboring molecules are handled in the short-range, many-body scheme; only interactions between molecules that are further apart are handled by the long-range scheme. Moving to *N*-meric modeling equates to expanding the dashed black boundary line in Figure 1 to include *N* molecules. This allows the truncation of the short-range, many-body scheme to be pushed further back in a systematic manner by moving from monomeric to dimeric modeling and beyond. In the case of water it has been shown^25^ that many-body effects beyond 3-body (where a body is a molecule) are relatively small. For example, the 4-body corrections to a water pentamer were only 2.1%. As such we expect that, in due course, at most a trimeric model will be required to model liquid water.

The problem of going from monomeric to dimeric modeling reduces to a machine learning problem. Whilst it is by no means a trivial problem, this treatment confers an important benefit in terms of computational cost. A dimeric model will be more expensive to train than a monomeric model because it will require more training points and each *ab initio* calculation is done on a larger system and so will be more expensive. However, the training is a one-off cost, which may involve a large and thus costly basis set but this cost has no trace in the model after training. The scaling of the computational cost of predicting with the GPR models is independent of the scaling (*e.g*., of O(*n*^7^) for CCSD(T) where *n* is a measure of the system size) of the calculations used to generate the training data. At the point of use in FFLUX, the predictions scale linearly with the total number of atoms in the simulation *N*_tot_, and the number of training points. Note that, while the number of training points will scale worse than linearly with the number of atoms described by the machine learning model, *N*_atom_, it is generally the case that *N*_atom_ ≪ *N*_tot_. Hence,the overall scaling is still linear with respective to the total number of atoms. This means that, despite including higher order many-body effects, the cost of predicting with a dimeric model scales no worse than with a monomeric model, up to a pre-factor. Even the worsening of the pre-factor is offset to some degree by the parallelization of the code. In other words, increasingly high-order many-body effects can be included with almost no detrimental effect on the scaling of the DL_FFLUX code. This applies equally to improving the level of theory used to compute the training data. These points are crucial to ensuring the long-term success of FFLUX.

The GPR models for each atom predict atomic multipole moments from charge (monopole) up to hexadecapole moments. As mentioned in the Introduction, these multipole moments come from the same QCT calculations as the short-range PES. As such, there is an underlying unity between the short-range electrostatics and the multipole moments that participate in the long-range electrostatics. These predicted multipole moments are fed into a classical smooth particle mesh Ewald (SPME) summation, resulting in long-range electrostatics that is rooted in quantum mechanics. The multipole moments, like the predicted energies, change as the geometry of a molecule changes. Hence they depend explicitly on atomic positions, that is, there is short-range polarization of multipole moments. The explicit dependence on position of multipole moments introduces extra force terms into the electrostatics. DL_FFLUX implements a modified version of SPME in order to allow for multipole moments of any rank that depend explicitly on atomic positions (so-called flexible multipole moments^26^). The van der Waals term in eq 3 is typically a Lennard-Jones or Buckingham potential (Lennard-Jones in this work).

Whilst the nature of the machine learning models introduces explicit short-range polarization, there is currently no explicit long-range polarization in FFLUX. This reveals the weakness of monomeric modeling, which is that there is only intramolecular polarization and no intermolecular polarization. However, given the well-known importance of intermolecular polarization, an effort is made here to capture this effect during the training of monomeric models. For each training point there are two calculations required to turn the input (atomic coordinates) into outputs (atomic energies and multipole moments). Firstly, a density functional theory (DFT) calculation is carried out using GAUSSIAN09^27^ to compute the wavefunction of the system. In order to include the effect of intermolecular polarization, the DFT calculation of the water monomer involves the addition of an implicit solvent. The atomic properties are then computed from the wavefunction using the program AIMAll^24^ that integrates over QCT atomic basins. The multipole moments learnt by the GPR model are then already polarized by an implicit solvent meaning that intermolecular polarization is implicitly accounted for in a FFLUX simulation.

To summarize, all short-range energies and forces in FFLUX are the result of a predicted many-body, *ab initio* PES. Long-range electrostatics is accomplished with high-rank multipole moments (in practice typically charges, dipole and quadrupole moments are used) that are predicted from *ab initio* calculations. These multipole moments are explicitly polarized by their short-range environment during a simulation and, in the case of monomeric modeling, are already implicitly polarized by their long-range environment. Both forms of polarization apply to multipole moments of all ranks. Polarization of anything beyond dipole moment is relatively uncommon even among modern force fields. However, we note that the NEMO potential includes polarization of quadrupole moments.^28^ The remaining long-range interactions are computed by a simple Lennard-Jones or Buckingham potential.

## 3 Technical Details

The GPR models used for water in this paper are monomeric models with just 100 training points. Initially, 50 random training points were used to generate a GPR model. The remaining points are then added *via* an iterative active learning procedure that ensures compact and efficient training sets. Full details can be found in reference.^21^ The DFT calculations were carried out at the B3LYP/aug-cc-pVTZ level of theory. This level of theory has been shown to perform well^29^ and was also chosen because the gas phase molecular dipole moment computed at this level of theory is 1.869 D, which agrees with the experimental value^30^ of 1.855 D within less than 1%. When combined with the implicit solvent, the molecular dipole moment is increased to 2.15 D, which is closer to the experimental value^31^ for liquid water of 2.9 ± 0.6 D. The integral equation formalism polarizable continuum model (IEFPCM) with the solvent set as water was used for the implicit solvent calculation in GAUSSIAN09.

**Figure 2:**
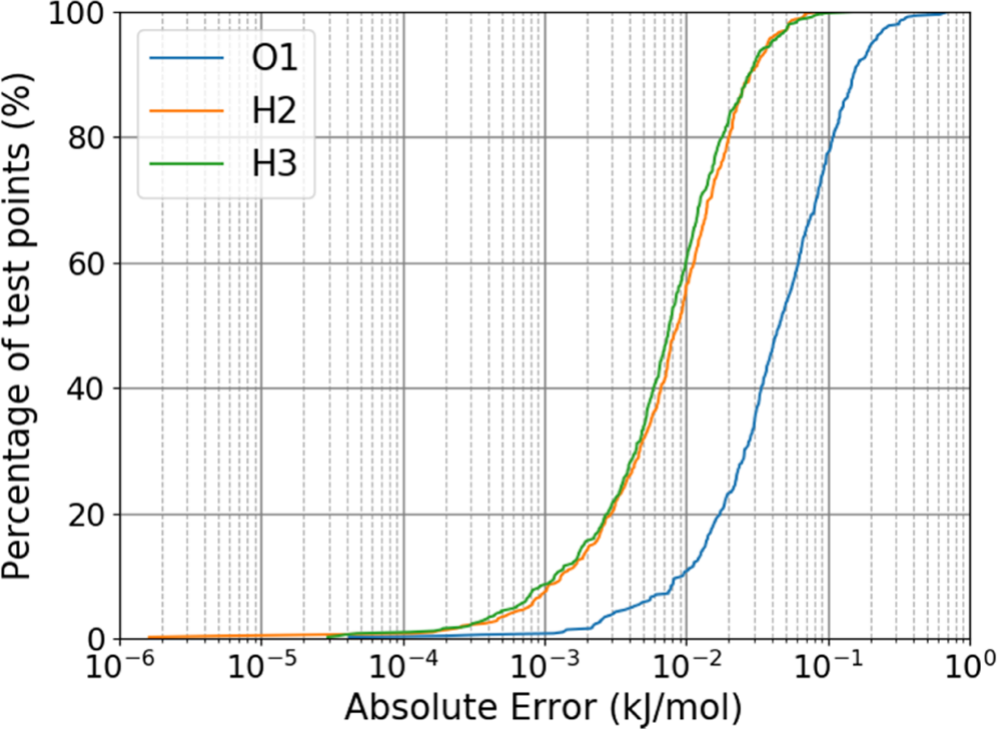
S-curve of absolute IQA energy prediction errors.

The GPR model is evaluated prior to being used in a simulation primarily using S-curves, which are cumulative error distributions so called because of their characteristic sigmoidal (“S”) shape. For a test set of 500 points (none of these points are in the training set), the predicted property is compared to the true value and an absolute prediction error is obtained. Figure 2 shows the S-curve of absolute prediction errors of each of the three atomic energies in kJ mol^–1^. The *y*-axis shows the percentage of the test points that have an error at or below a given prediction error in kJ mol^–1^. Almost all of the points have an error of less than 0.1 kJ mol^–1^ (80% for O) and all of the points are predicted to within less than 1 kJ mol^–1^. The model predicts to well within chemical accuracy (usually taken to be 1 kcal mol^–1^) with just 100 training points.

The charge predictions perform similarly well for this model as shown in Figure 3. Almost all of the points are predicted to within 1 millielectron (me) accuracy and the majority are within 0.1 me. The S-curves for all components of the dipole and quadrupole moments can be found in the Supporting Information (SI), Figures S1–S8.

**Figure 3:**
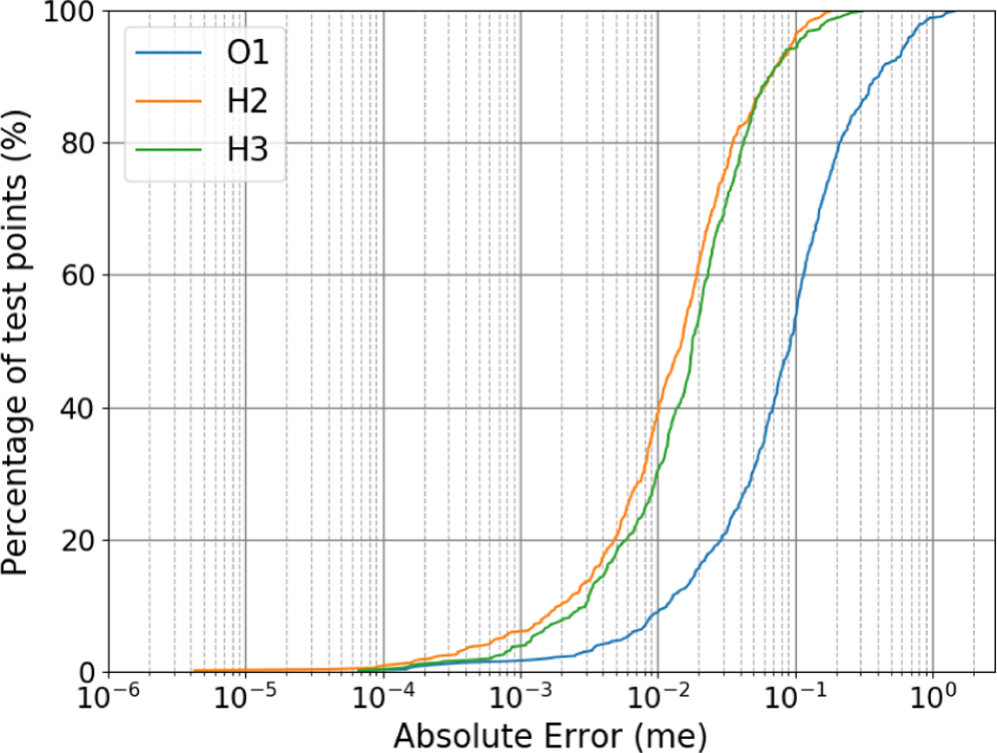
S-curve of absolute charge prediction errors.

Simulations were performed in the NPT ensemble using the Nosé–Hoover barostat. The timestep was 1 fs and a cut-off radius of 8 Å was used for all simulations. Unless stated otherwise, each run was 2.5 ns in length. At each temperature 10 simulations were run: 4 with a box of 216 molecules, 3 with a box of 343, and 3 with a box of 512 molecules. However, in the case of the simulations performed at 320, 330, and 340 K, a total of five simulations were carried out for a box of 343 molecules. This smaller number of simulations is due to the fact that the diffusion coefficient was not computed at these temperatures. Also, at 298 K extra simulations were performed for a large box of 1728 molecules (4 runs) as well as 3 extra runs for each of the 343 and 512 molecule boxes. In total, liquid water was simulated at 11 different temperatures. The starting configurations for each trajectory were taken from the endpoint of a 1 ns simulation. The initial velocities were randomly scaled in order to generate multiple different trajectories from the same initial configuration. The parameters for the SPME Ewald sum were determined automatically using DL_POLY with the keyword “spme precision 1d-7”.

In all simulations monopole, dipole and quadrupole moments were enabled and all interactions up to and including quadrupole-quadrupole computed. This is denoted *L*′ = 2 where the 2 corresponds to the highest rank multipole moment enabled. Note that in our previous QCT-based simulations^32−34^ on liquid water, albeit with rigid water geometries (using the program DL_MULTI^35^), we used a different type of multipolar interaction governed by  where  refer to the rank of an atomic multipole moment (*e.g*.,  for a dipole moment). In our previous work *L* was set to 5, which means that 3 extra interaction terms were included compared to *L*′ = 2: monopole–octupole, monopole–hexadecupole and dipole–octupole (and the reverse, *i.e*., octupole–monopole, *etc*.). The multipole moments used in DL_FFLUX are traceless Cartesian moments in the global frame. Either traced or traceless moments can be used providing the appropriate pre-factors are included with the moments (this is only a consideration for quadrupole moments and beyond). The Lennard-Jones parameters shown in Table 1 were found by running on the order of 100 simulations with various parameters, perturbed from an initial parameter set taken from previous work,^34^ and inspecting the density of each simulation. In particular, the OH parameters are identical while the OO parameters were taken as a starting guess and ended up with values quite similar to the ones used in that work. The parameter combinations were reduced to six promising candidates that were found to produce densities close to the experimental value. The diffusion coefficient in the infinite box size limit was estimated for these six candidates and the set of parameters that gave the best estimated value was chosen. Note that DL_FFLUX has recently been parallelized with domain decomposition MPI. More information on timings is available in reference.^36^

**Table 1:** Lennard-Jones Parameters Used for Liquid Water Simulations

parameter	value
ε_OO_	0.763 kJ mol^–1^
σ_OO_	3.17 Å
ε_OH_	0.106 kJ mol^–1^
σ_OH_	1.902 Å

## 4 Results and Discussion

### 4.1 Assessment of Electrostatics

Figures 2 and 3, alongside Figures S1–S8, in the Supporting Information give an indication of how well the GPR models are predicting. However, in the case of multipole moments (especially those of higher rank than monopole) these curves are of limited utility. It is difficult to have much intuition about, for example, the error in a given component of a quadrupole moment. As such, we developed a method to study the errors associated with the predictions of multipole moments in a manner that offers more insight. This method assembles the various interactions between multipole moments into a corresponding electrostatic energy, the prediction error of which then serves as a physically meaningful summary for the prediction errors in the multipole moments themselves.

**Figure 4:**
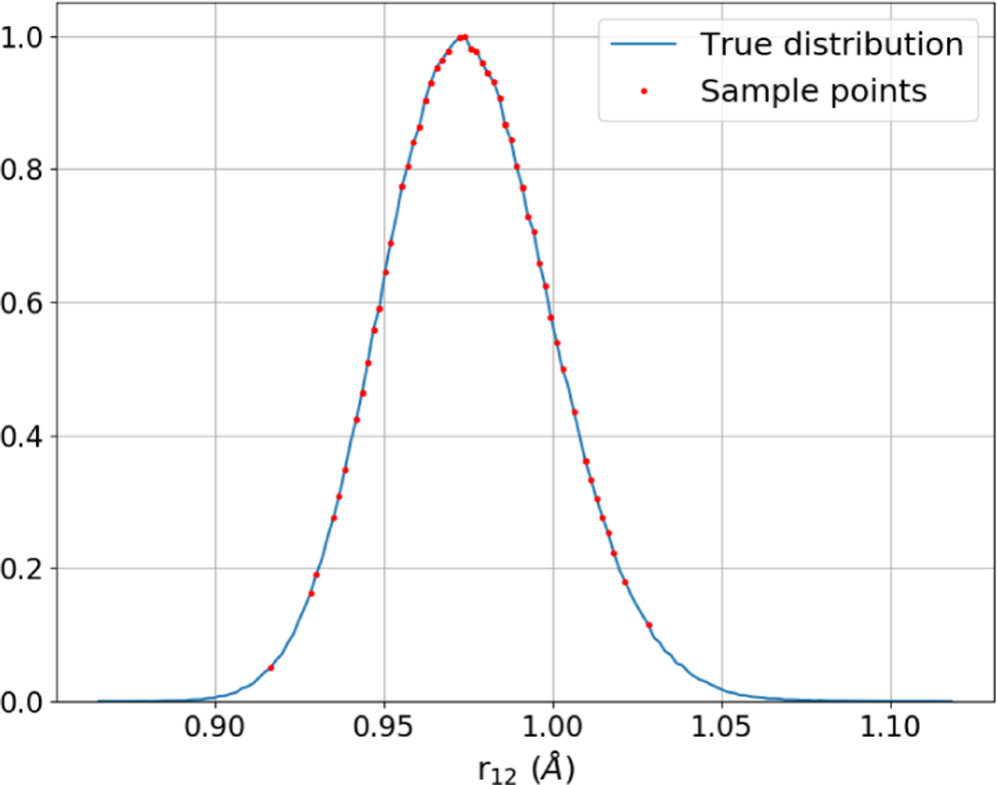
Distribution of O–H bond lengths from a 100 ps liquid water simulation. The bond lengths in the 100 randomly sampled dimers are shown by red circles.

An in-house program called PROMETHEUS was used to analyze a 100 ps trajectory from a FFLUX water simulation. The program collected all pairs of water molecules at every timestep with an oxygen–oxygen distance of 3.5 Å or less. The water dimer is a 6-atom system and so requires 12 (=3 × 6 – 6) dimensions in order to fully describe its geometry. Each of the water molecules is described by 2 O–H bond lengths and 1 H–O–H angle, totaling six dimensions that describe all intramolecular degrees of freedom. Thus the remaining six dimensions are used to specify the relative geometries of the two molecules. One of the water molecules is used to define a local frame while the relative position and orientation of the other water is then specified using spherical polar coordinates for its oxygen and three Euler angles (defined in this local frame) for its orientation. By analyzing these dimers, PROMETHEUS generates distributions for each of these 12 dimensions that are then randomly sampled to produce 100 dimers that are a good representation of the dimers seen in a real simulation. An example of one of the O–H bond length distributions is shown in Figure 4, which demonstrates that the 100 sample points span almost the full range of bond lengths seen in a simulation. These 100 dimers are then subjected to a test to determine how well the electrostatics is performing *in situ.*

**Figure 5:**
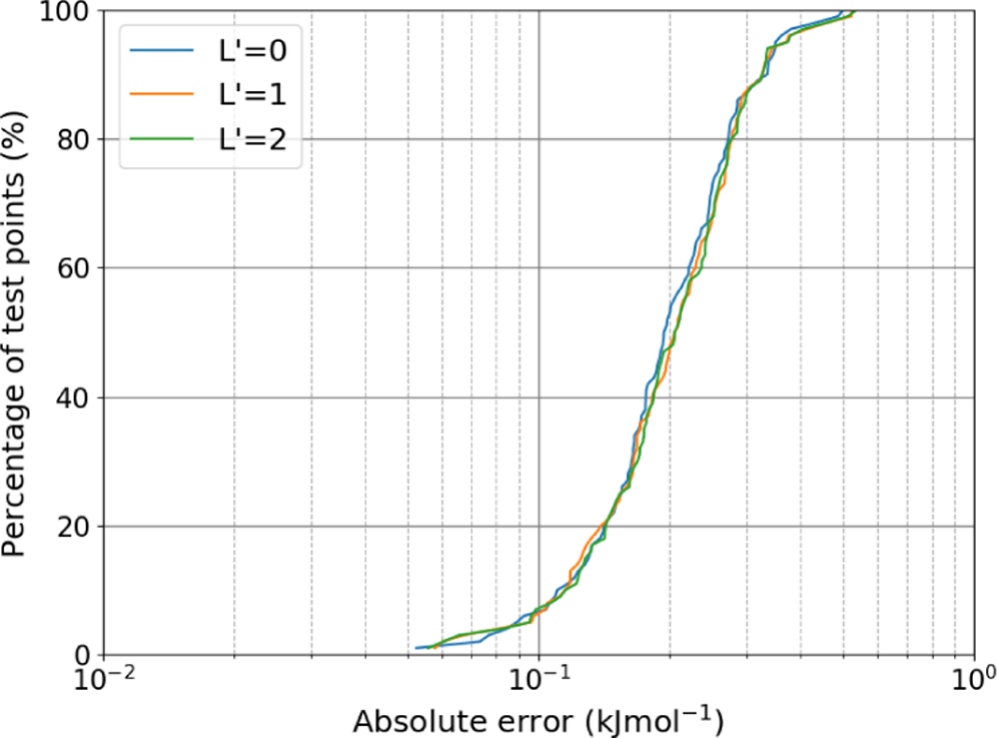
S-curves showing the absolute electrostatic errors in the electrostatics for 100 water dimers.

For each dimer, the GPR models are used to predict the multipole moments for each atom, which are then used to compute the electrostatic energy. This is the predicted energy, which must then be compared against the “true energy”. In order to obtain this energy, the DFT and then QCT calculations are carried out on each dimer to get the “true” multipole moments that are then used to compute electrostatic energies. Note that, in order to for the true-versus-predicted test to be a like-for-like comparison, the wavefunction and then multipole moments of each molecule in the dimer must be computed separately. This is because the models are monomeric, and so do not include explicit intermolecular polarization. Note that the convergence of the multipole expansion has been studied in considerable detail in the past.^17,18^ Performing these tests on the 100 dimers allows us to produce a new S-curve, shown in Figure 5.

The S-curve shows the test carried out at the levels of the electrostatics: *L*′ = 0, 1 and 2. At *L*′ = 0, only charge–charge interactions are computed, while at *L*′ = 1, dipole moments are switched on, and *L*′ = 2 is the level used for all simulations in this work, which utilizes monopole, dipole and quadrupole moments. For a given dimer geometry, the errors shown in Figure 5 are computed as follows: the electrostatic interaction between each of the atom pairs (a total of nine pairs: O1–O4, O1–H5, ...) is computed with the true and predicted multipole moments. For each of these nine interactions, the absolute difference between the true and predicted is taken and then summed to produce a total electrostatic error for the dimer. This is the error that is then plotted in Figure 5. It is evident that the success shown in

Figures 2 and 3 is replicated *in situ*, where it really matters. Summing the absolute errors makes this test very stringent and yet, almost all of the dimers have a total error of less than 0.5 kJ mol^–1^. All levels of the electrostatics perform similarly well, suggesting that the GPR models are able to predict monopole, dipole and quadrupole moments with high accuracy. A further examination of convergence of the electrostatics is given in the Supporting Information (Figures S9–S11).

### 4.2 Summary of Bulk Properties

Table 2 summarizes the bulk properties of liquid water at 298 K that are studied in this paper. More detail is given about each of the properties in the relevant section. Properties are also compared to a selection of four force fields that were mentioned in the introduction: MB-Pol, HIPPO, SIBFA21 and AMOEBA+(CF). These were chosen as they provide a good overall representation of the current state- of-the-art. Where a property is not shown for a particular force field it is because it is not given.

**Table 2:** Summary of Properties of Liquid Water at 298 K for FFLUX and Various Force Fields^a^

property	AMOEBA+(CF)^b^	MB-Pol	SIBFA21	HIPPO^b^	FFLUX	experiment
ρ (kg m^–3^)	998.4	1007	996.1	996.5	996.7	997.0^37,38^
ρ_max_ (K)	281.15^d^	263	265^d^	277	285.9	277.15^37,38^
*D* (10^–9^ m^2^ s^–1^)	2.14	2.34	1.47	2.56	1.93	2.30^39^
Δ*H*_vap_ (kJ mol^–1^)	44.35	45.73	49.41	43.81	41.94	43.93^38^
*C*_P_ (J mol^–1^ K^–1^)	87.45^c^	117.15	116.98	85.27	129.67	75.31^38^
α (10^–4^ K^–1^)	2.5	3.7		2.75	1.93	2.6^40^
ε_r_	78.8	68.4	79.03	76.9	34.5	78.4^38^

a Data for AMOEBA+(CF),^7^ MB-Pol,^29^ SIBFA2^14^ and HIPPO^5^ taken from references given.

b Data given at 298.15 K.

c Value given at 303.15 K.

d This value is not quoted in the corresponding paper but deduced by ourselves from their tabled data without us fitting their data.

### 4.3 Radial Distribution Functions (RDFs)

**Figure 6:**
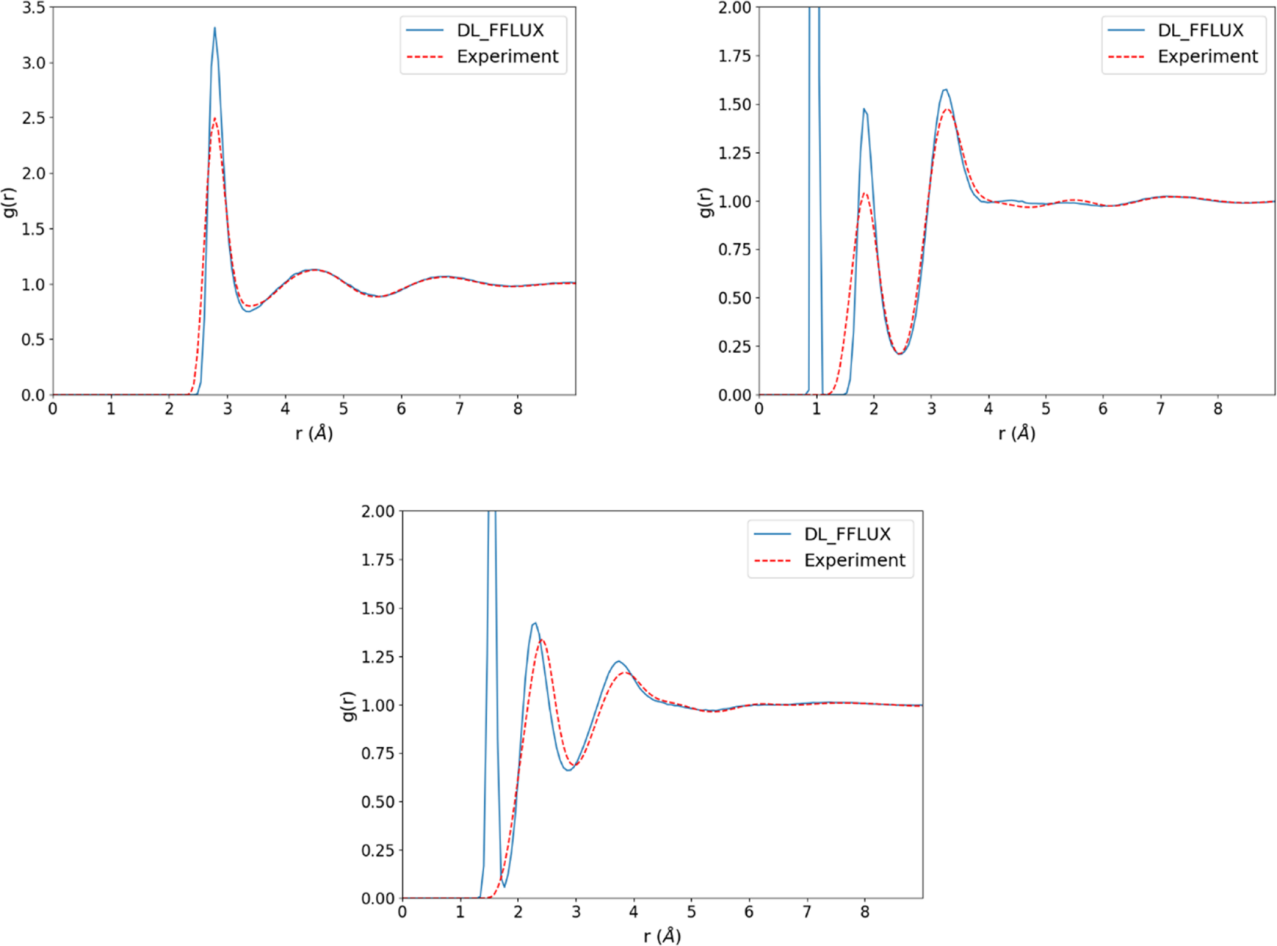
Radial distribution functions (computed using VMD^42^). Top left: oxygen–oxygen, top right: oxygen–hydrogen and bottom: hydrogen–hydrogen. Experimental data taken from Soper.^43^ Note that the simulated radial distribution functions include intramolecular contributions whereas the experimental data do not.

The FFLUX RDFs shown in Figure 6 agree very well with experiment in terms of peak positions. For the oxygen–oxygen and oxygen–hydrogen RDFs, the positions of the peaks are essentially perfect and for the hydrogen–hydrogen RDF, there is a slight shift to the left. In all cases, the peak heights are larger than experiment. The RDFs for the other force fields are not shown here but can be found in the relevant references. In general, all four force fields predict the RDFs very well. However, just as for FFLUX, all of the other force fields exhibit varying degrees of overpredictions of the peak heights. MB-Pol attributes this in part to a lack of NQE^41^ whereas AMOEBA+^1^ suggests that using a Buckingham potential rather than a Lennard-Jones could improve the RDFs. Consistent with previous work,^32^ we found that the radial distribution functions are sensitive to the electrostatics. For simulations with point charges only, the RDFs were hugely overstructured (*i.e*., peaks are far too large). We also found that, when a model was used that had been trained without implicit solvation, there was essentially no structure in the radial distribution functions. We therefore conclude that some degree of polarization is necessary to recover the correct structure of liquid water. This finding complements previous work^32^ simulating liquid water with fixed QCT multipole moments (*i.e*., no polarization). In that work it was found that there was little structure in the RDFs unless octopole and hexadecapole moments were included (up to charge–hexadecapole interactions). That observation, taken together with the findings in this work, suggests that including multipole moments beyond quadrupole can offset some of the detrimental effects of not accounting for polarization.

### 4.4 Diffusion Coefficient

**Figure 7:**
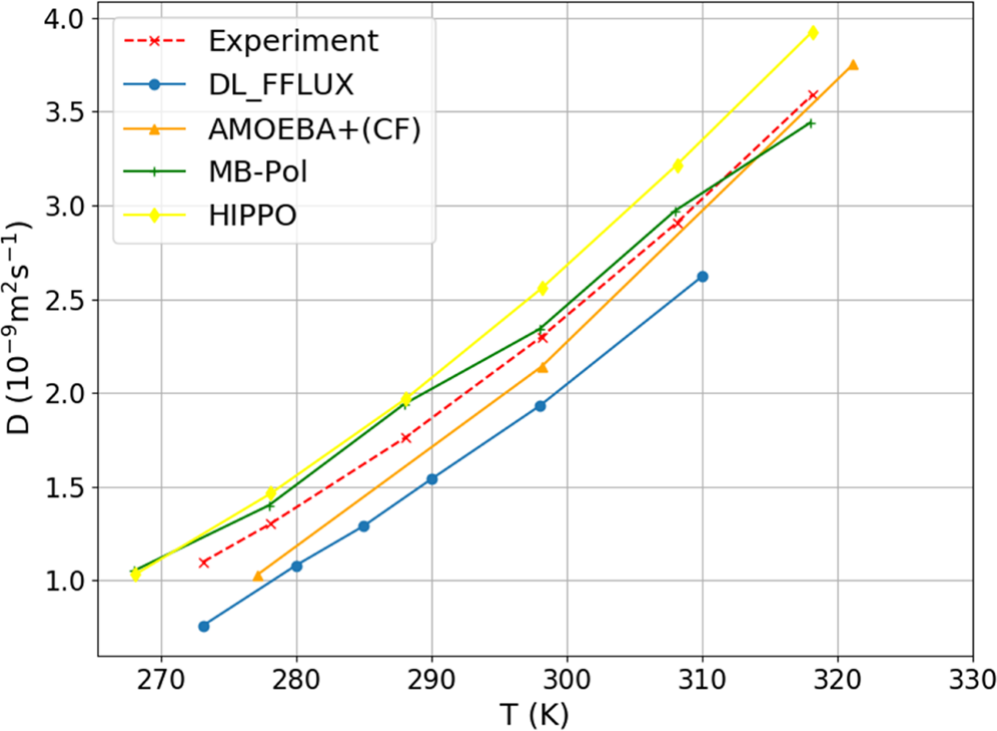
Diffusion coefficient computed at various temperatures with FFLUX and other force fields. Experimental data taken from reference.^39^

The diffusion coefficient can be computed from the Einstein relation,
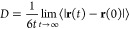
4where **r**(t) and **r**(0) are position vectors at time *t* and *t* = 0, respectively. The diffusion coefficient is known to depend on the size of simulation performed^44^ and it is now the standard to take this into account. As such, at each of the three box sizes (216, 343 and 512 molecules), an ensemble average over trajectories was carried out in order to obtain a value for the size-dependent diffusion coefficient, *D*(L). Each *D*(*L*) was then corrected using eq 5 in order to retrieve the diffusion coefficient in the infinite size limit,^44^
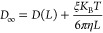
5where *D*_∞_ is the diffusion coefficient in the infinite size limit, η is the viscosity (the experimental viscosity was used here) and ξ = 2.837297 for a cubic box with side length *L*. Note that there was some variability in the infinite size limit diffusion coefficient predicted by each of the box sizes. At each temperature, the average of the *D*_∞_ computed at each of the box sizes was taken. This is the value plotted for each point in Figure 7. The uncertainty in the diffusion coefficient (reported in Table S1) is calculated as the root mean square deviation between the average diffusion coefficient and the values for each of the box sizes.

In order to investigate the validity of eq 5, an additional four 2.5 ns simulations were conducted at 298 K for a much larger box containing 1728 water molecules. This enabled an alternate method of computing *D*_∞_. A plot of *D*(*L*) *vs* 1/*L* was made and fitted to a straight line. The *y*-intercept of the fit is then the infinite size limit diffusion coefficient (see Figure S12). The diffusion coefficient computed in this way was 1.91 × 10^–9^ m^2^ s^–1^,which is very close to the value of 1.93 × 10^–9^ m^2^ s^–1^ computed using eq 5. The excellent agreement (within 1%) between the two methods validates the application of eq 5.

The FFLUX diffusion coefficient curve in Figure 7 is consistently below the experimental curve by a constant amount over the whole temperature interval calculated. Unlike the AMOEBA+(CF) profile the DL_FFLUX profile is shifted, which means that the latter’s slope is the same as the experimental one. The diffusion coefficient is well known to be impacted by nuclear quantum effects (NQE). The inclusion of NQE has been shown^45^ to increase the value of the diffusion coefficient by 15–53%. The fact that the trend in Figure 7 matches experiment well is encouraging as it suggests that the underprediction will be alleviated by accounting for NEQ. Conversely, force fields that already overpredict (such as HIPPO) the diffusion coefficient will not be helped by including NQE.

### 4.5 Liquid Density

**Figure 8:**
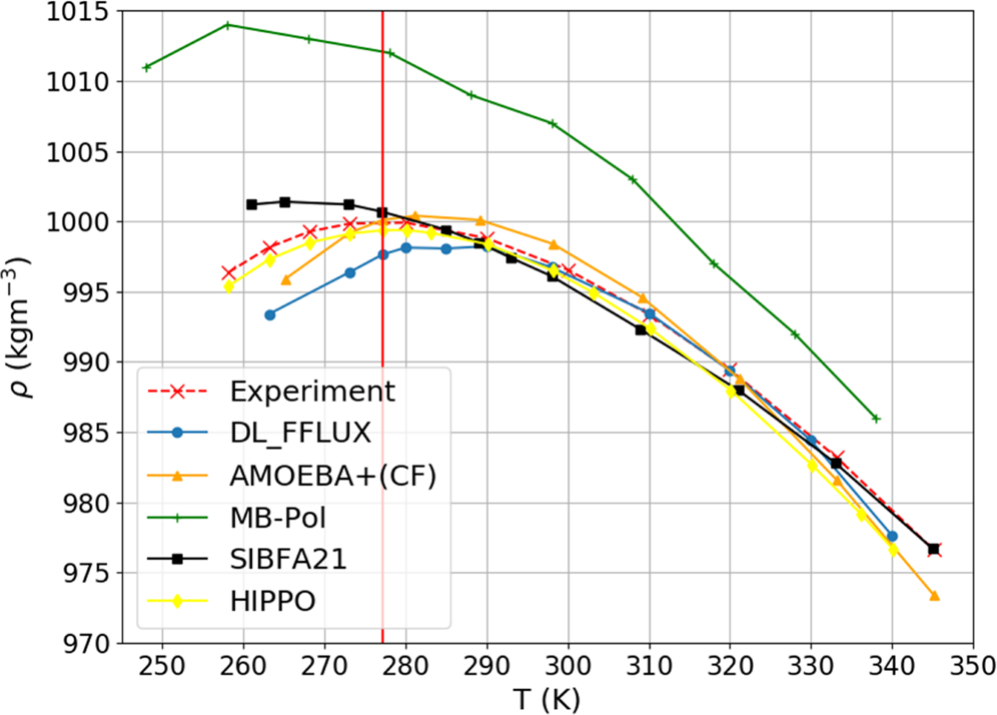
Density of liquid water computed at various temperatures with FFLUX and other force fields. Experimental data taken from references.^37,38^

The density curve was fitted to a third order polynomial and analytically differentiated in order to find the maximum density. Figure S13 shows this fit, which makes clear that there is indeed a maximum between 280 and 290 K, which does not visually appear in Figure 8. This figure shows how the density of liquid water varies with temperature as predicted by FFLUX, simply by linking the data points rather than fitting them to a polynomial. We find that the maximum appears at 285.9 K (based on 10 2.5 ns simulations), which overpredicts the experimental value (of 277.15 K) by 8.75 K. Note that MB-Pol underpredicts this value by 14.15 K while HIPPO predicts it spot on. The convergence of the ensemble averaging was examined using leave-one-out cross-validation. The total average was compared to the average when one trajectory is removed from the ensemble. This was done for all trajectories and the root mean square of these deviations taken as the uncertainty. A well converged property will change very little when one trajectory is removed from the average. Conversely, a poorly converged property will change considerably. Note that the error bars are too small to be seen in Figure 8 but the values are given in Table S2 in the Supporting Information.

Figure 8 shows the dependence of the liquid’s density on temperature. The FFLUX density curve generally agrees well with experiment. Significant deviation from experiment only starts to occur at temperatures below 290 K. The most likely explanation for this deviation is that the Lennard-Jones parameters were optimized for the correct density of liquid water at 298 K. As the water cools and crystallizes, these parameters are no longer suitable. This is a clear limitation of monomeric modeling although we note that FFLUX is not alone in performing worse at lower temperatures, as shown in Figure 8. Here we see that MB-Pol’s value is the furthest removed from the experimental one, which the authors claim results from the absence of NQE. At the highest temperatures, only SIBFA21 follows experiment very well while the other force fields start underpredicting.

### 4.6 Thermal Expansion Coefficient

**Figure 9:**
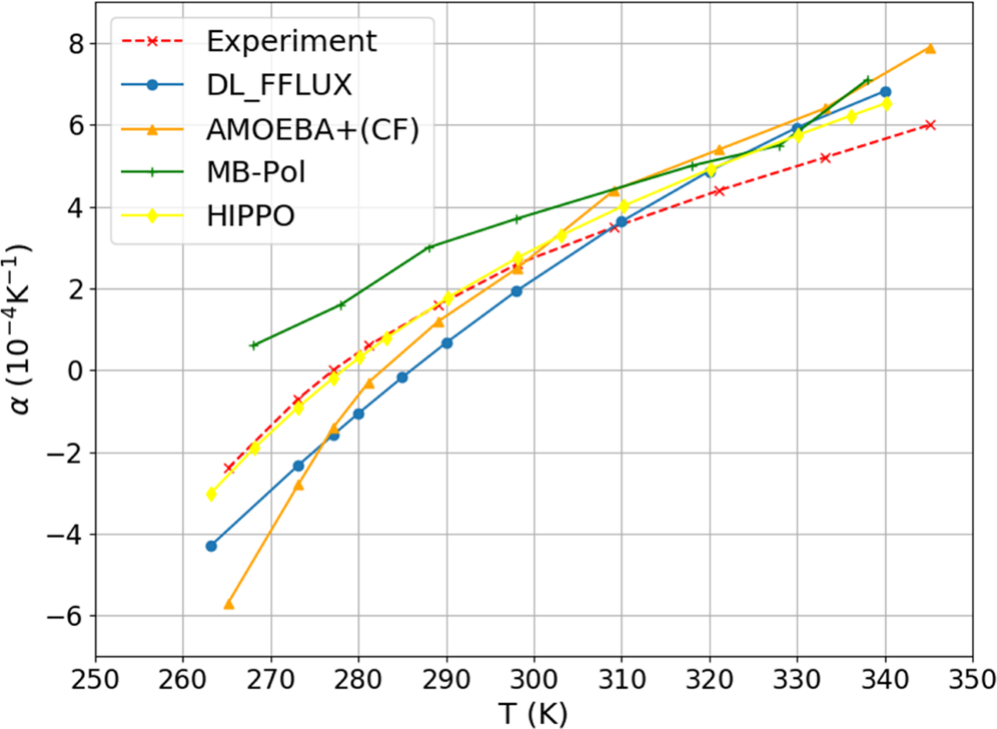
Thermal expansion coefficient computed at various temperatures with FFLUX and other force fields. Experimental data from reference.^40^

The thermal expansion coefficient α can be computed according to the following equation,
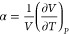
6where *V* is volume. Note that α can be negative when either d*T* is positive and d*V* is negative (*i.e*., increasing temperature decreases the volume), or d*T* is negative and d*V* is positive (*i.e*., decreasing temperature increases the volume). The density data from Figure 8 were turned into a *V*(*T*) curve, which was then fitted to a third order polynomial. This polynomial was then analytically differentiated in order to produce the curve α(*T*)shown in Figure 9. The FFLUX curve is steeper than the experimental curve, which suggests that the volume responds more than it should to a change in temperature, *i.e*., heating the water leads to a larger than expected increase in volume. This is an indication that the FFLUX liquid water behaves more like a gas than the true liquid. This trend is consistent with what we see later with the enthalpy of vaporization and is most likely a result of the limitations of monomeric modeling. Indeed, despite the inclusion of implicit solvation, monomeric modeling is still rooted in the gas phase.

The comparison between force fields is shown in Figure 9, where HIPPO follows experiment almost spot on below about 300 K. MB-Pol overpredicts more than any other force field below about 310 K but is then joined by the other force fields in terms of overprediction above 310 K.

### 4.7 Enthalpy of Vaporization

The enthalpy of vaporization, Δ*H*_vap_, is the difference between the gas and liquid phase enthalpy as per eq 7,
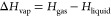
7

**Figure 10:**
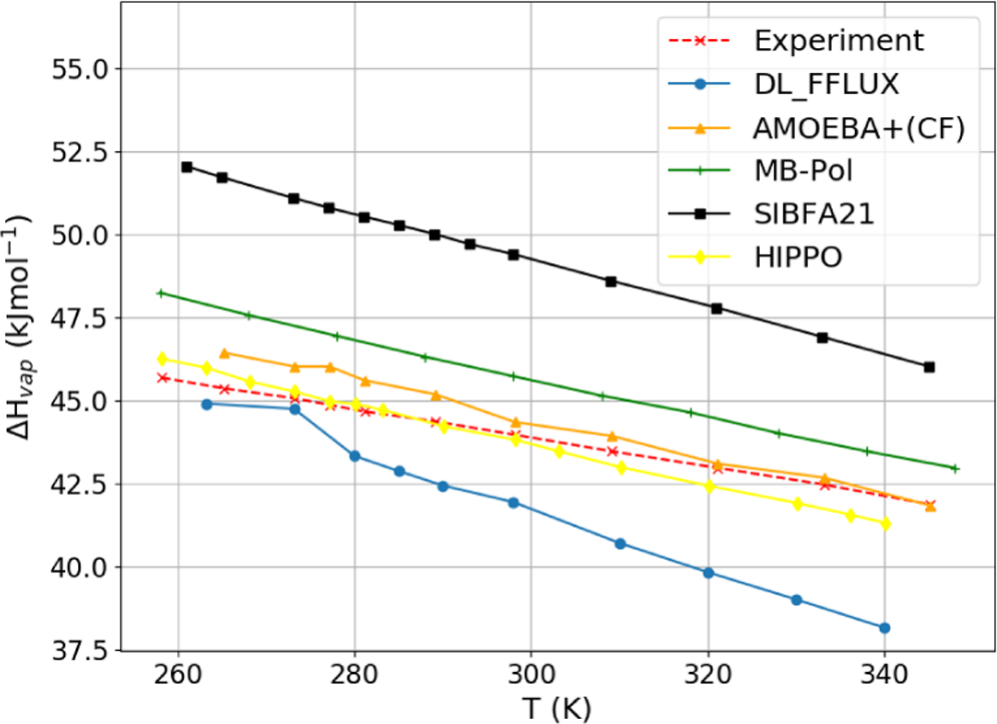
Comparison of enthalpy of vaporization curves computed with various force fields. Experimental data taken from reference.^38^

The gas phase enthalpy was computed from 250 ps simulations of water monomers at various temperatures. In absolute terms, the FFLUX enthalpy of vaporization curve in Figure 10 is relatively close to experiment with a maximum deviation of less than 1 kcal mol^–1^. However, the FFLUX curve is consistently below the experimental curve, *i.e*., the difference in enthalpy between the gas and the liquid phase is smaller than it should be. This means that it is easier than it should be to turn FFLUX’s liquid water into a gas. This is unsurprising given the limitations of monomeric modeling. The effects that are missing in the case of monomeric modeling are stabilizing effects. Without them, it is natural that the resulting liquid is closer to a gas than the real liquid. This also explains why the FFLUX curve decreases more quickly than it should as temperature increases. Indeed, as temperature increases, the liquid water approaches the gas phase more quickly than it should, *i.e*., FFLUX liquid would likely boil at a lower temperature than real liquid water. This has a knock-on effect on the isobaric heat capacity as seen in the next section. The steeper gradient of the curve in Figure 10 is driven by a larger increase in the liquid enthalpy as temperature increases, which results in an overprediction of the heat capacity.

### 4.8 Isobaric Heat Capacity

**Figure 11:**
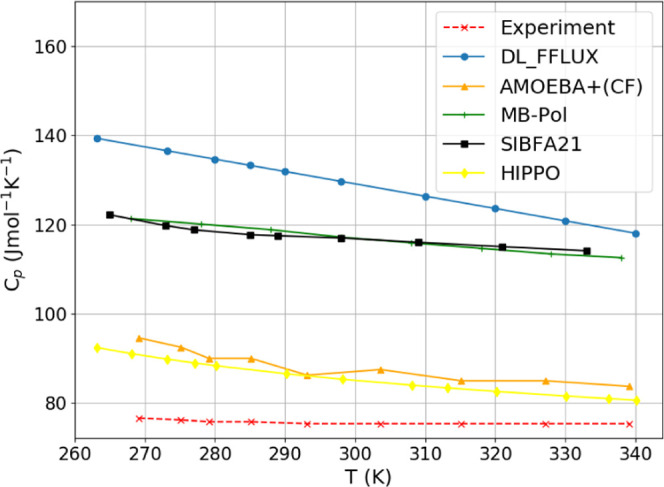
Comparison of isobaric heat capacity curves computed with various force fields. Experimental data taken from reference.^38^

The isobaric heat capacity is computed by fitting the liquid enthalpy *vs* temperature curve to a second order polynomial and then differentiating with respect to temperature per eq 8,
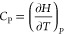
8where *H* is the liquid enthalpy. Figure 11 shows that FFLUX considerably over predicts the isobaric heat capacity. Nuclear quantum effects are known to reduce *C*_P_. However, as discussed in the previous section, this overprediction is also a consequence of the fact that the enthalpy versus temperature curve is steeper than it should be regardless of NQE. As such the FFLUX overprediction is a result of a combination of a lack of NQE and the limitations of monomeric modeling.

### 4.9 Infrared Spectrum

The infrared (IR) spectrum is computed by taking the Fourier transform of the autocorrelation function of the total system dipole moment,
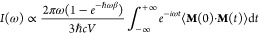
9where ω is a frequency and **M** is the total system dipole moment vector and β = 1/*K*_B_*T*. The expression in angular brackets in eq 9 denotes the autocorrelation function. The total system dipole moment is the sum of molecular dipole moments **μ**, which themselves are comprised of atomic dipole moments and charge transfer dipole moments. Note that it is necessary to multiply eq 9 by a quantum correction factor.^46^ It has been reported that this choice is somewhat arbitrary.^47^ In this work we have chosen the factor that gives the best IR spectrum although we found that all of the choices performed essentially identically (see the Supporting Information for more information). Note that dipole data were printed every timestep (every 1 fs). Printing intervals of 0.5 fs were tried but there was no appreciable difference in the IR spectrum.

**Figure 12:**
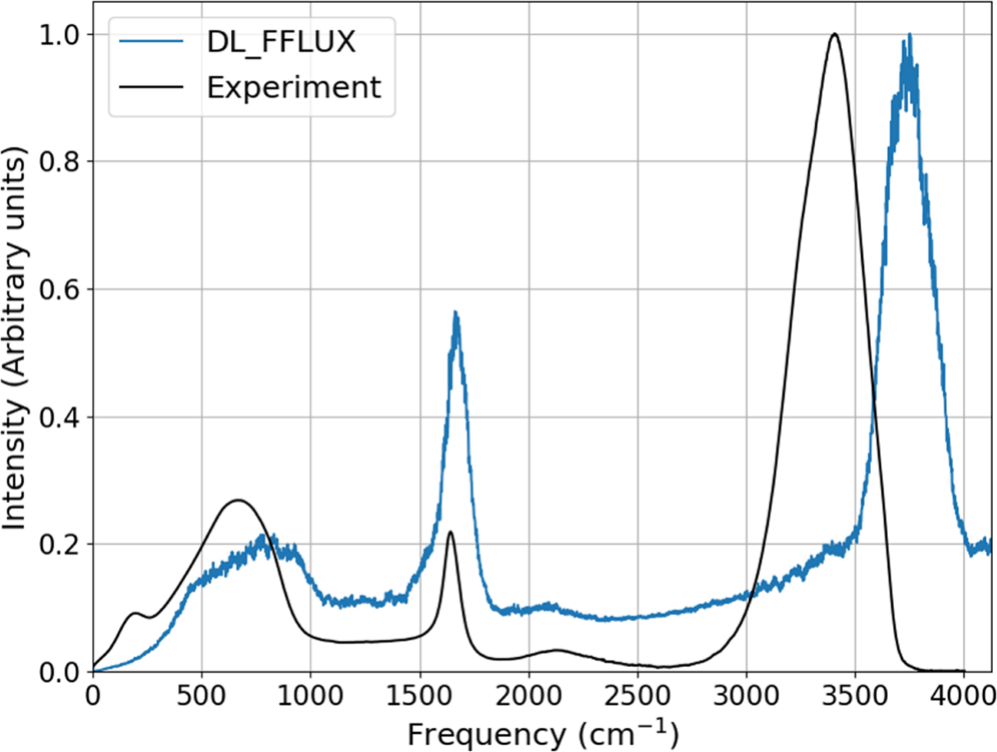
Comparison of the FFLUX and experimental infrared spectra. Experimental data taken from reference.^48^

The intensity in both the simulated and experimental data is normalized such that the maximum is 1. This is to allow easier comparison of peak heights. The IR spectrum in Figure 12 is generally in good agreement with experiment. However, there are two key features of the IR spectrum that FFLUX gets wrong. Firstly, the peak at roughly 200 cm^–1^ that corresponds to collective intermolecular vibrations is missing in the FFLUX spectrum. This is known to be a peak that only emerges with the inclusion of many-body polarization effects (where body here corresponds to a molecule).^8,49^ Secondly, the OH stretching peak is shifted to higher frequencies. This is an effect that has been studied in recent work with the MB-Pol potential,^8^ which shows that in order to remove this shift, one must include many-body effects as well as NQE. We note that the AMOEBA+(CF) force field sees a shift of this peak to the correct place relative to the AMOEBA+ force field. However, this shift is due to a tweaked force constant designed for exactly this purpose.

### 4.10 Relative Permittivity

The relative permittivity is computed according to the following equation,
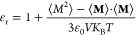
10where the brackets denote ensemble averages and ε_0_ is the permittivity of free space. Over a long enough time, the average of the total system dipole moment vector, ⟨**M**⟩ will go to zero and eq 10 reduces to eq 11. In our own calculations the contribution from this term was close enough to zero such as to be ignored. The relative permittivity was computed from three 2.5 ns trajectories of a box of 216 water molecules. The data to compute the total system dipole were printed every 10 timesteps (every 10 fs).
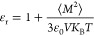
11

FFLUX predicts a value for the relative permittivity of 34.5 at 298 K, which is a large underprediction relative to the experimental value of 78.4. This is also considerably worse than any of the other force fields shown in Table 2. The relative permittivity is the property that most highlights the shortcomings of monomeric modeling. The lack of intermolecular polarization with monomeric modeling means that the molecular dipole moments in FFLUX are on average only 2.15 D. This is higher than the gas phase but, according to previous work,^50^ is only at about the level expected from a water molecule positioned in a trimer. This is considerably less than the experimental value of 2.9 ± 0.6 D for liquid water, which leads to the low value for ε_r_. Importantly, the aforementioned previous work shows that a water molecule placed in a large enough cluster (see Figure 3 in ref (50)) will have a molecular dipole moment of 2.3–2.5 D, which is closer to the experimental value. This shows that QCT does in fact attain the correct molecular dipole moment if intermolecular polarization is accounted for.

In order to assess our explanation of the underprediction of the relative permittivity, a test was carried out. At every timestep, the raw molecular dipole moment vectors were scaled by a constant factor in order to make the average magnitude ∼2.5 D. Note this was done in post-processing, not during a simulation. This scaling led to a calculated relative permittivity of 57.0. This test confirms that the relative permittivity is highly sensitive to the magnitude of the molecular dipole moments. However, this is only half the story because the relative alignments of the molecular dipole moments matter when computing the total system dipole moment. A proper treatment of intermolecular polarization would lead not only to larger magnitudes of molecular dipole moments but also to moments that are better aligned, further increasing the magnitude of the total system dipole moment and thus the relative permittivity.

## 5 Conclusions

As water is the most studied liquid it is not surprising that, over several decades, dozens of potentials have been developed for it by many groups. If one can loosely speak of generations of potentials then a first generation would be confined to point charges and without polarization, such as the popular SPC and TiPnP (*n* = 3, 4, or 5) family. The majority of these potentials restrict water to be rigid but flexibility can be added by harmonic bonded potentials. Second generation potentials, such as AMOEBA, SIBFA, MB-Pol and HIPPO, would then include atomic multipole moments (for improved electrostatics) and polarizabilities. FFLUX shares the idea of atomic multipole moments but is not rooted in intermolecular perturbation theory. Instead, FFLUX starts from the atomic partitioning of matter, both for energies and moments, where the piece of matter can be a single molecule or a cluster thereof. FFLUX uses a quantum topological energy partitioning that starts from the electron density and reduced density matrices. As such FFLUX “sees the electrons” and is aware of the internal energy of an atom. FFLUX uses machine learning (Gaussian process regression) to learn the relation between an atom’s energy and the geometry of all the other atoms in the system. FFLUX thus recovers flexibility in a natural way and accounts for all electronic effects in a molecule. In the case of water this ability accounts for H···H interactions (unusually) but in more complex molecules it captures all effects, no matter how subtle and no matter their absence in earlier generation force fields. The architecture of FFLUX thus introduces atoms that interact with each other without a distinction between being bonded or non-bonded. FFLUX is generally applicable beyond water and maintains the strategy of using only *ab initio* data for as long as possible. It may be argued that some of the above features start shaping a third generation of force fields.

As far as current testing goes, FFLUX is able to successfully predict atomic energies and multipole moments, in a single coherent scheme, for molecules of up to 30 atoms and allowing for generous molecular distortions. In this work we present the first simulation of a box of such predictive (Gaussian process) models, in which the multipolar electrostatics works alongside energy fluctuations both within the atoms, as between the atoms of a monomer. This monomer modeling (of water) forces the non-electrostatic intermolecular energy to be modeled by a temporary but most familiar device: a four-parameter Lennard Jones potential. Incidentally, in currently ongoing work, the same FFLUX technology is being used to model a formamide crystal, using non-bonded parameters. Future work will eliminate externally added non-bonded potentials by *N*-meric modeling where the machine learning takes care of the non-electrostatic intermolecular energy in a more sophisticated, fully integrated and unified way.

With monomeric modeling we looked at the maximum liquid density as a function of temperature, the (self)-diffusion coefficient, the vaporization enthalpy, the isobaric heat capacity, the thermal expansion coefficient, the relative permittivity and the IR spectrum. Where FFLUX is wrong one can argue that it is wrong for the right reasons. In other words, issues like the missing low frequency peak in the IR spectrum and the underpredicted relative permittivity are direct consequences of the approximate nature of monomeric modeling. These approximations are well understood and there is a clear path forward to improving these approximations by moving to dimeric and eventually *N*-meric modeling. Crucially, the architecture of FFLUX allows this transition to higher accuracy to happen without a need to overhaul the force field and without introducing worse computational scaling. Still, as the comparison currently stands we believe that the force field FFLUX finds a place amongst the other state-of-the-art force fields for liquid water simulations. Moreover, as is, the FFLUX methodology can also be used for modeling aqueous solvation of small to medium-sized molecules.

## Supporting Information Available

Additional S-curves: Figures S1–S3 for dipole moments; Figures S4–S8 for quadrupole moments. Note on time steps. Electrostatic convergence: Figures S9–S11; diffusion coefficient: Table S1 (dependence on temperature, *T*), Figure S12 (dependence on box size); liquid density: Table S2, Figure S13 (*T*-dependence); thermal expansion coefficient: Table S3 (*T*-dependence); enthalpy of vaporization: Table S4 (*T*-dependence); isobaric heat capacity: Table S5 (T-dependence); infrared spectrum; distributions of monomeric properties: Figure S14 (O–H bond length); Figure S15 (H–O–H angle); and radial distribution functions: O–O, O–H, H–H (PDF)
