# Supplementary material for: Application of Quantum Chemical Topology Force Field FFLUX to Condensed Matter Simulations: Liquid Water
Source: J Chem Theory Comput. 2022 Aug 8;18(9):5577–88. doi: 10.1021/acs.jctc.2c00311 (PMC9476653; doi:10.1021/acs.jctc.2c00311)
Supplement: Supplementary file 1 — ct2c00311_si_001.pdf [file ct2c00311_si_001.pdf]

Supporting Information

Application of quantum chemical topology force field FFLUX to condensed matter simulations: liquid water

Benjamin C. B. Symons and Paul L. A. Popelier

Additional S-curves

This section gives the S-curves for the 3 components of the dipole moment and the 5 components of the quadrupole moment. Note that the multipole moments are learnt and predicted in spherical tensor form. Subsequently they are converted to Cartesian tensors inside the program DL_FFLUX. As such the S-curves are given for the components in spherical tensor form.

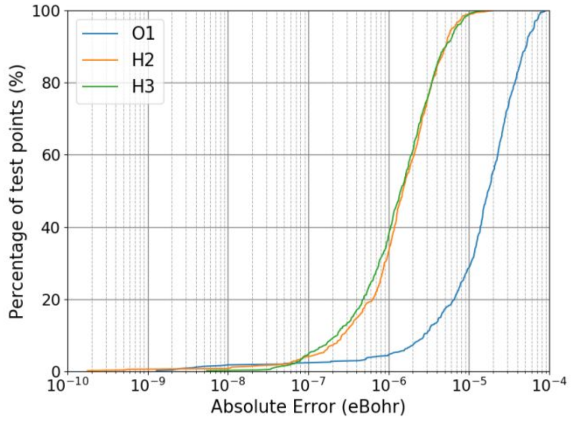

Figure S1. S-curve for the Q_{10} component of the dipole moment.

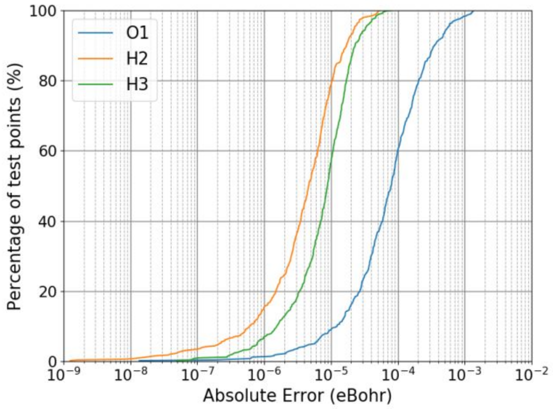

Figure S2. S-curve for the Q_{11c} component of the dipole moment.

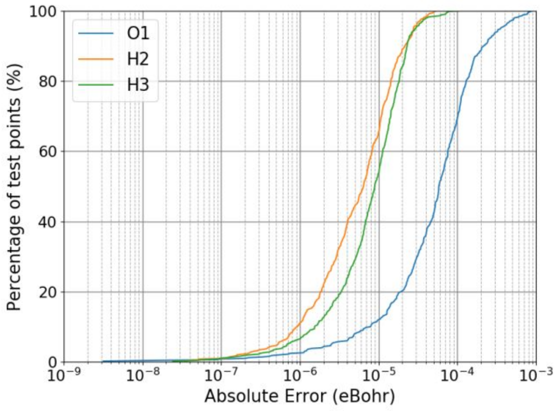

Figure S3. S-curve for the Q_{11s} component of the dipole moment.

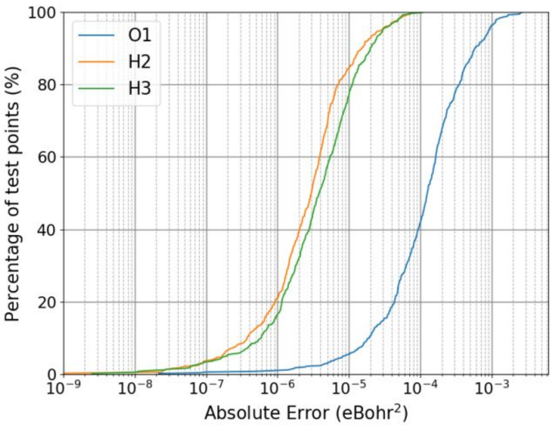

Figure S4. S-curve for the Q_{20} component of the quadrupole moment.

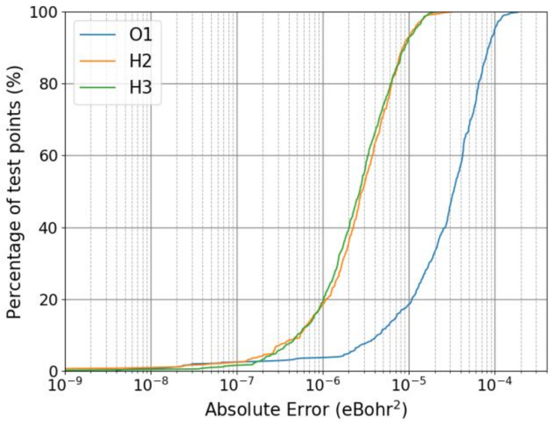

Figure S5. S-curve for the Q_{21c} component of the quadrupole moment.

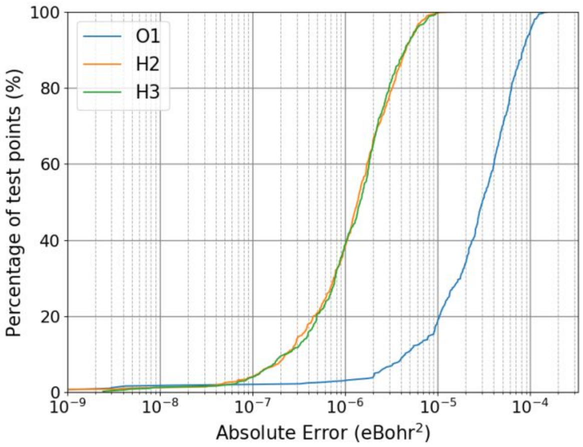

Figure S6. S-curve for the Q_{21s} component of the quadrupole moment.

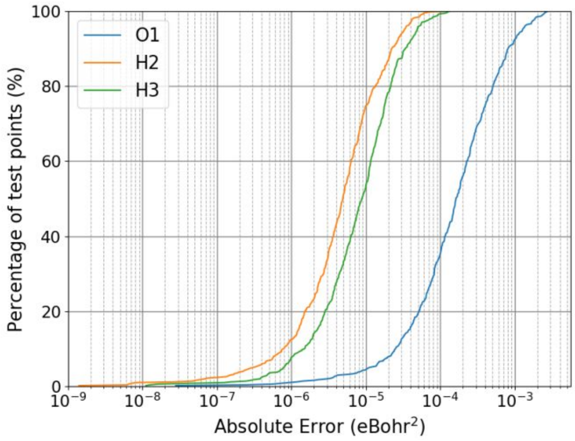

Figure S7. S-curve for the Q_{22c} component of the quadrupole moment.

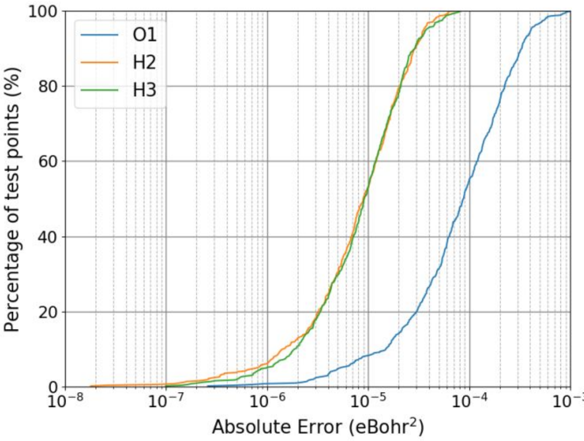

Figure S8. S-curve for the Q_{22s} component of the quadrupole moment.

Note on time steps

The timestep chosen for DL_FFLUX runs was 1 fs, which represents a middle ground compared to the timesteps used by the four other force fields that we compare with, ranging from 0.2 fs to 2 fs. In particular, properties obtained with AMOEBA+ using 0.5 fs and 2 fs differ little, while our own test on the IR spectrum using 0.5 fs showed an improvement that was too marginal to justify the computational cost. We note that MB-pol uses 0.2 fs, which is very small, while SIBFA21 uses 1 fs and HIPPO 2 fs. Very few properties are known to be overly senstitive to timestep but researchers often reduce the timestep from say 1 fs to 0.5 fs for the IR spectrum.

Electrostatic convergence

Convergence has two meanings in the context of the multipole expansion. The first relates to the formal mathematical condition that, if satisfied, ensures the expansion is well defined and thus guaranteed to converge. If this condition is violated (this may happen when charge distributions overlap) then the expansion may diverge. The other kind of convergence relates to the truncation of the infinite series at a finite number of terms. A study of this kind of convergence examines how the series approaches the infinite limit value (the 'true' value) as an increasing number of terms are included. A considerable amount of work has been done examining this kind of convergence[1-3]. The results presented here build on the work of Rafat and Popelier[4], which examines the convergence of the multipole expansion in the case of a water dimer in the geometry of the global energy minimum. Specifically the hydrogen bond O-H interaction was studied in refernce 4. This work served as a useful benchmark during the development of the DL_FFLUX code.

Figure 1 of reference 4 (Rafat and Popelier) is a key plot that is partially (up to L=5) reproduced in Figure S9 with some added data of our won. Figure S9 shows the electrostatic energy of the hydrogen bond in the water dimer at various values of $L=l_A+l_B+1$ where l_A and l_B are the rank of the multipole moments on a pair of interacting atoms *A* and *B,* respectively. L determines which electrostatic interactions are calculated, e.g. at L=2 charge-charge and charge-dipole interactions are calculated while L=5 includes terms up to charge-hexadecapole. The horizontal red line in Figure S9 is the true value of the electrostatic interaction, which is determined via a 6-dimensional integral carried out by the program AIMAll. All of the data (multipole moments, geometries etc.) were plugged into DL_FFLUX in order to check whether DL_FFLUX reproduced the curve of Rafat and Popelier. This was a highly important validation step as the two curves, that overlap perfectly in Figure S9, are produced by entirely different computer programs. This test is a crucial validation of the DL_FFLUX code.

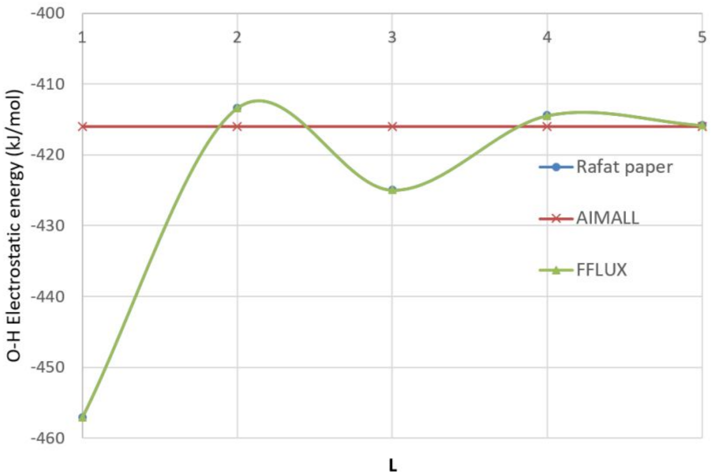

Figure S9. Reproduction of part of Figure 1 from Rafat and Popelier[4]. The red horizontal line is the result of the 6D integration using the program AIMAll. The blue curve is taken from the Rafat paper (data truncated at L=5). The FFLUX curve was produced using the same inputs as Rafat but using the DL_FFLUX code.

The work of Rafat and Popelier in Figure S9 shows that relatively high rank multipole moments (octopole and hexadecapole) are required for the expansion to converge well. In order to determine whether or not this finding generalises across many dimer configurations, not just the global minimum, another test using the in-house program PROMETHEUS was carried out. This test used the same 100 water dimers as described in the main text. The convergence of the multipole expansion was assessed by comparing the 6D integration energy of the electrostatic interaction between each pair of atoms in the dimer (9 pairs in total) to the energy as computed using the multipole expansion. The multipole moments that are fed into the multipole expansion are computed from the dimer geometries as follows. Firstly, a wavefunction for each dimer was computed using DFT at the B3LYP/aug-cc-pVTZ level of theory with the program GAUSSIAN09. The wavefunction was then fed to the program AIMAll, which computes the atomic multipole moments. In other words, QCT multipole moments are fed into the expansion and compared against the QCT true energy. The expansion is truncated at various points and the difference between the true and expansion energies computed.

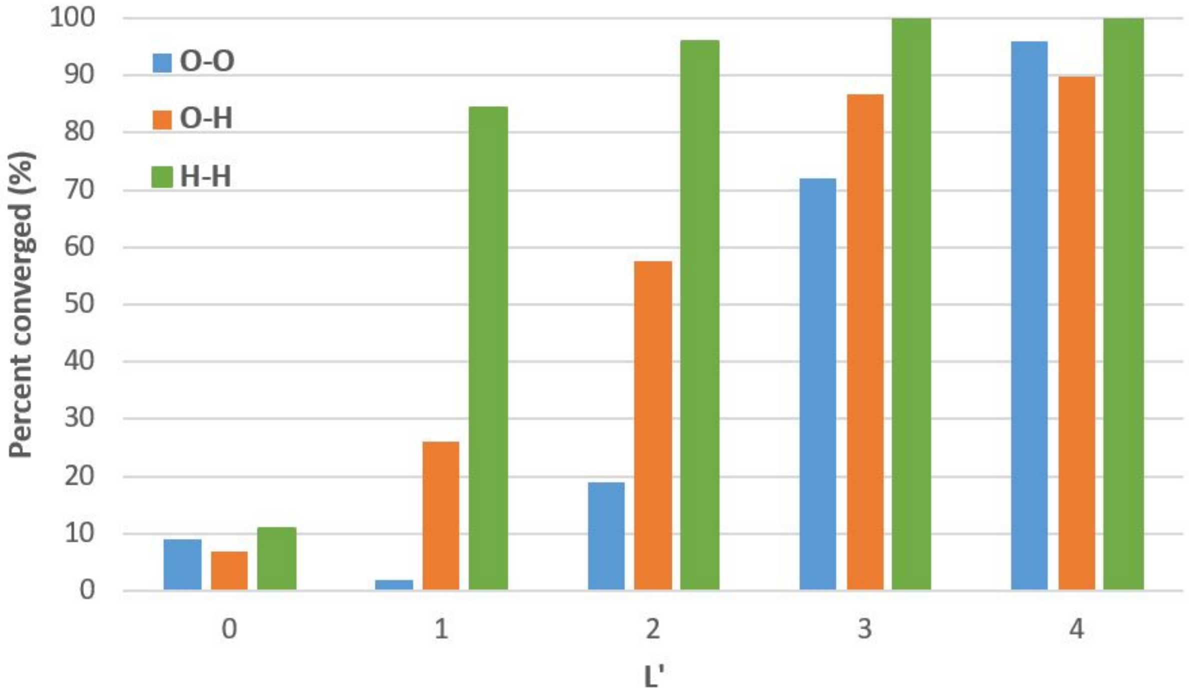

Figure S10. Percentage of oxygen-oxygen (O-O), oxygen-hydrogen (O-H) and hydrogen-hydrogen (H-H) interactions in 100 water dimers that have converged to within 1 $kJmol^{-1}$ of the true electrostatic energy at five possible values of L'.

Figure S10 shows how well the multipole expansion converges at various values of L'. Note that we are now using the L' convention rather than the L convention mentioned above. A convergence criterion of 1 kJ/mol at such short range is a stringent test. The higher the rank of the moments involved the interaction, the more quickly the term dies off as a function of distance. The proportionality to distance is R^{-L}. As such, the short range is where the higher order terms will still be relevant. The largest allowed oxygen-oxygen distance in the 100 dimers is 3.5 Å (chosen as it encompasses the first solvation shell) which is very much short range. Figure S11 shows the same graph with the convergence criterion relaxed to 1 $kcalmol^{-1}$.

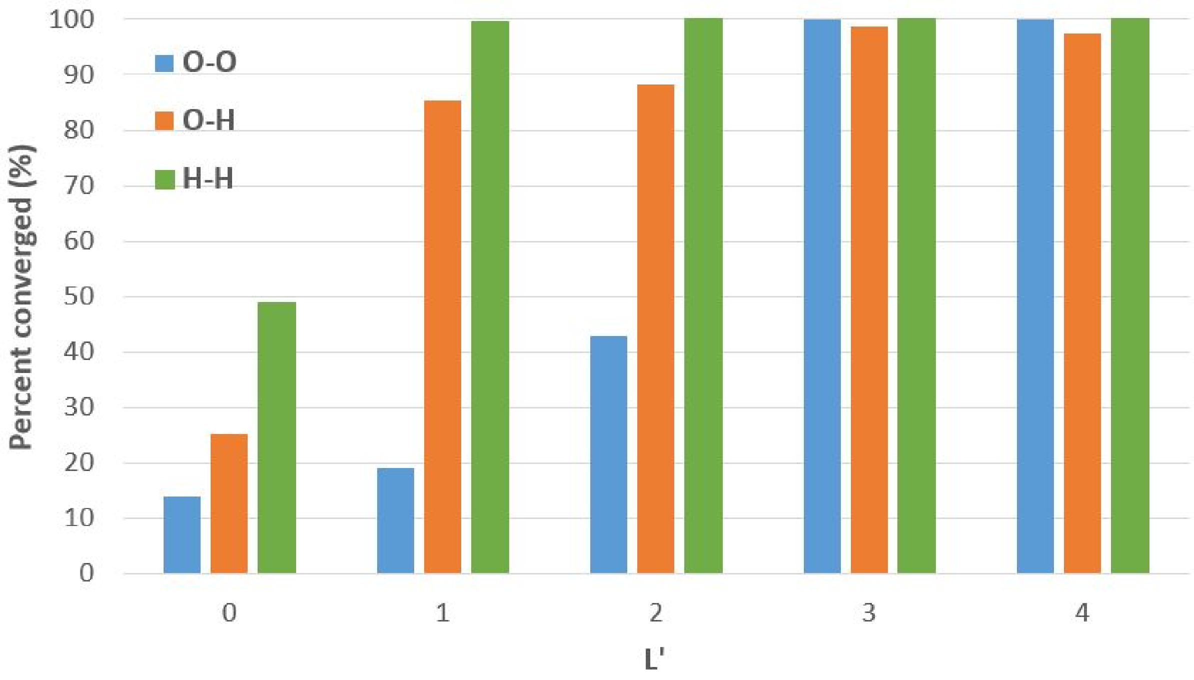

Figure S11. Percentage of oxygen-oxygen (O-O), oxygen-hydrogen (O-H) and hydrogen-hydrogen (H-H) interactions in 100 water dimers that have converged to within 1 kcalmol^{-1} of the true electrostatic energy at five possible values of L'.

In both Figures S10 and S11 it is evident that hydrogen atoms are better described than oxygen atoms by lower rank multipole moments. This is unsurprising given there is considerably less electron density on hydrogen atoms. It is also clear that, even with a convergence criterion of 1 kcalmol^{-1}, the electrostatics is poorly described at the level of point charge only. In order to get a good description of short-range electrostatics, at minimum dipole and quadrupole moments are essential. If near perfect convergence is the goal then even octopole moments are required. However, this comes at considerable computational cost and arguably may not be worth the gain. This work shows that the findings of Rafat and Popelier[4] generalise to configurations of the water dimer beyond just the global minimum. These findings are in line with the push towards including high rank multipole moments in many state-of-the-art force fields.

Diffusion coefficient

Temperature (K)	Diffusion coefficient (10^{-9} m^2 s^{-1})
273.15	0.76 (0.06)
280	1.08 (0.02)
285	1.29 (0.02)
290	1.54 (0.02)
298	1.93 (0.02)
310	2.62 (0.01)

Table S1. Diffusion coefficient predicted by FFLUX at various temperatures. Values in brackets are uncertainties.

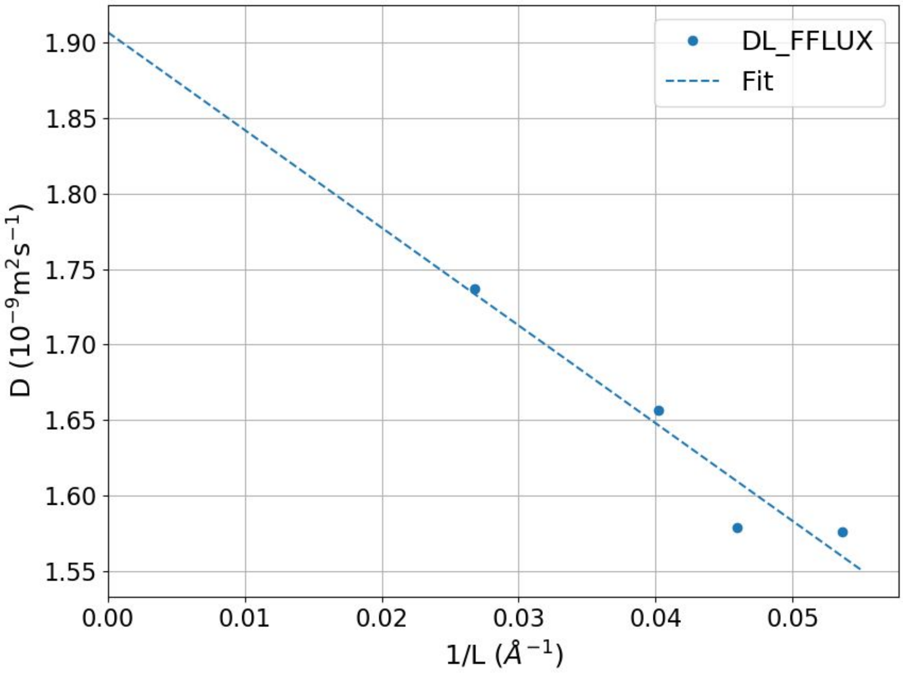

Figure S12. Plot of diffusion coefficient against inverse box length at 298 K. Dashed line shows the fit extrapolated to the infinite box size limit.

Figure S12 demonstrates the alternative method for computing the diffusion coefficient mentioned in the main text. This method requires simulations to be carried out with a sufficient number of different size boxes. The extra runs done with larger boxes (1728 molecules) at 298 K enable a comparison of the two methods of computing the diffusion coefficient. The method in Figure S12 results in a value that deviates, from that by the method in the main text, by only 0.02 units (1.03 %).

Liquid density

Temperature (K)	Density (kg m^{-3})
263.15	993.40 (0.35)
273.15	996.40 (0.14)
277.15	997.64 (0.10)
280	998.13 (0.09)
285	998.07 (0.05)
290	998.22 (0.05)
298	996.73 (0.03)
310	993.48 (0.10)
320	989.38 (0.08)
330	984.40 (0.14)
340	977.63 (0.22)

Table S2. Density predicted by FFLUX at various temperatures. Values in brackets are uncertainties.

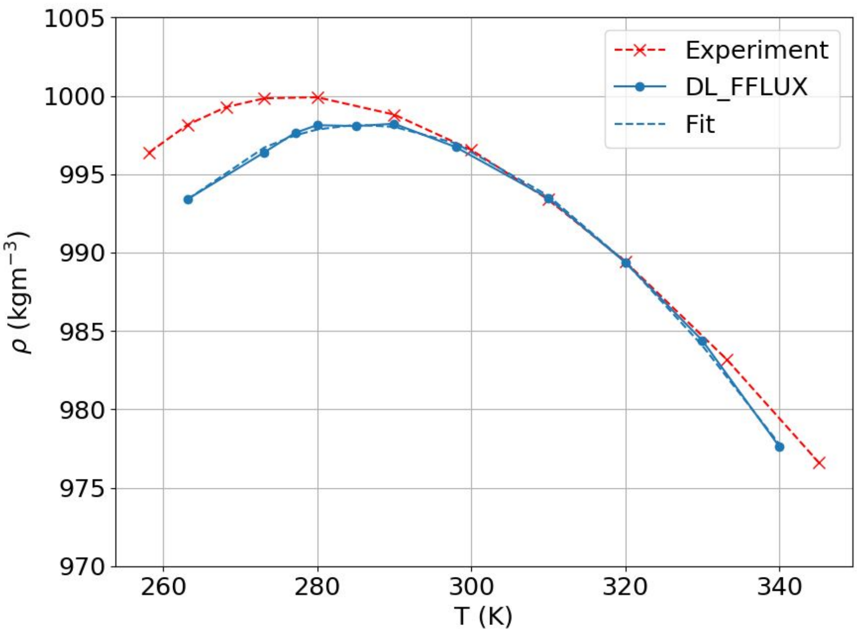

Figure S13. Plot showing the FFLUX density curve as well as the 3rd order polynomial fit (dashed blue line). Figure S13 demonstrates that the 3rd order polynomial is an excellent fit to the data.

Thermal expansion coefficient

Temperature (K)	Thermal expansion coefficient (10^{-4} K^{-1})
263.15	-4.30
273.15	-2.33
277.15	-1.58
280	-1.06
285	-0.18
290	0.67
298	1.93
310	3.64
320	4.87
330	5.93
340	6.82

Table S3. Predicted thermal expansion coefficient of FFLUX at various temperatures.

Enthalpy of vaporisation

Temperature (K)	Enthalpy of vaporisation (kJ mol^{-1} K^{-1})
263.15	44.91
273.15	44.75
280	43.33
285	42.88
290	42.44
298	41.94
310	40.71
320	39.83
330	39.01
340	38.16

Table S4. Predicted enthalpy of vaporisation of FFLUX at various temperatures.

Isobaric heat capacity

Temperature (K)	Enthalpy of vaporisation ($Jmol^{-1}K^{-1}$)
263.15	139.32
273.15	136.55
280	134.66
285	133.27
290	131.89
298	129.67
310	126.35
320	123.58
330	120.81
340	118.05

Table S5. Predicted isobaric heat capacity of FFLUX at various temperatures.

Infrared spectrum

As reported in the main text, there are several choices for the quantum correction factor. These are shown in Equations S1, S2 and S3. The exact choice is relatively arbitrary and it is generally the case that the factor that produces the best spectrum is chosen. We find in this work that all of the factors give essentially the same spectrum.

$$2/(1+\exp[-\beta \hbar \omega]) \quad \text{(S1)}$$

$$\frac{\beta \hbar \omega}{1-\exp[-\beta \hbar \omega]} \quad \text{(S2)}$$

$$\exp[\beta \hbar \omega / 2] \quad \text{(S3)}$$

Distributions of monomeric properties

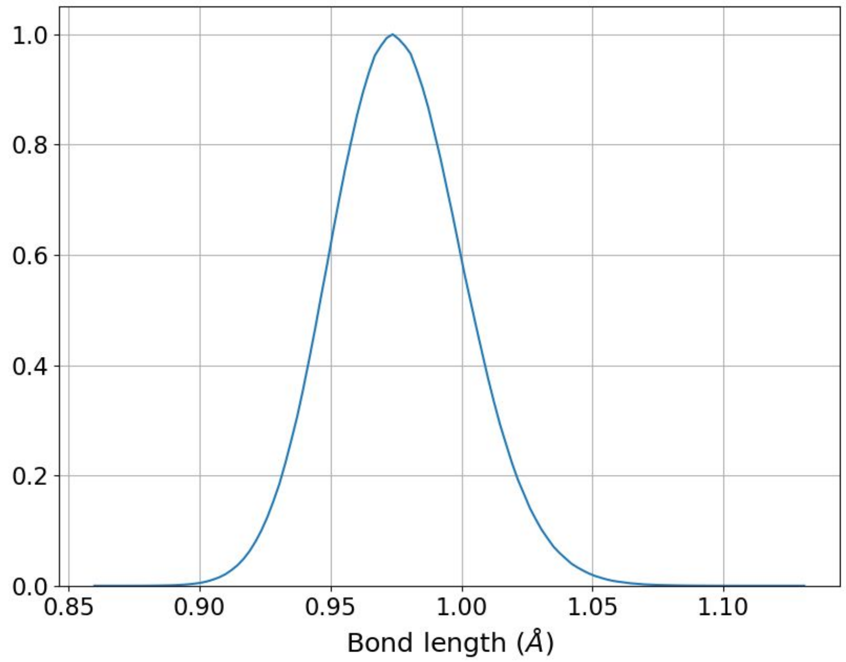

Figure S14. Distribution of O-H bond lengths in liquid water.

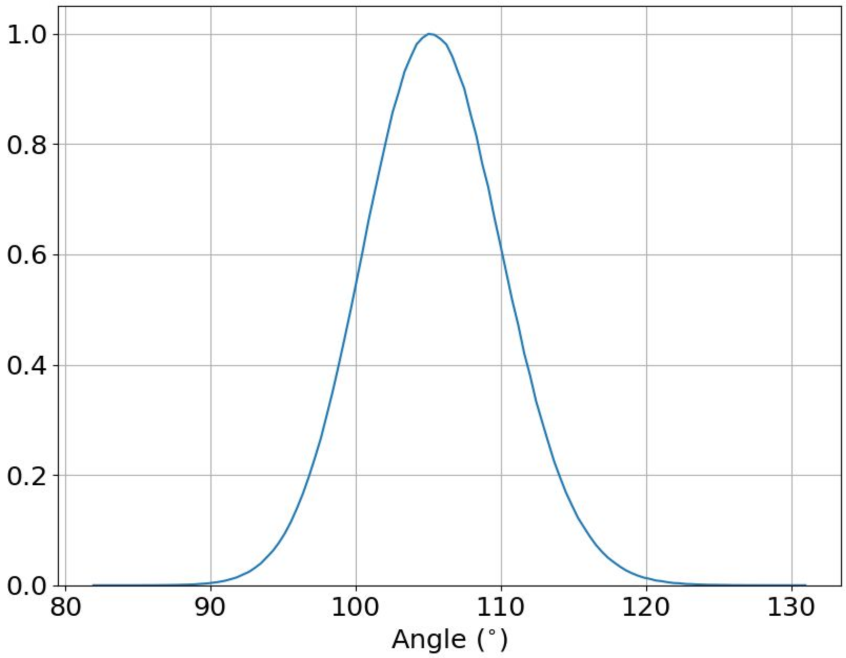

Figure S15. Distribution of H-O-H angles in liquid water.

Radial distribution functions

The following sections provide the raw data for each of the RDFs plotted in the main text.

Radial distribution function: Oxygen-Oxygen

r (A)	g(r)
0.03	0
0.09	0
0.15	0
0.21	0
0.27	0
0.33	0
0.39	0
0.45	0
0.51	0
0.57	0
0.63	0
0.69	0
0.75	0
0.81	0
0.87	0
0.93	0
0.99	0
1.05	0
1.11	0
1.17	0
1.23	0
1.29	0
1.35	0
1.41	0
1.47	0
1.53	0
1.59	0
1.65	0
1.71	0
1.77	0
1.83	0
1.89	0
1.95	0
2.01	0
2.07	0
2.13	0
2.19	0
2.25	0
2.31	0
2.37	0
2.43	3.10E-05

2.49	0.006272
2.55	0.114611
2.61	0.686612
2.67	1.848884
2.73	2.9573
2.79	3.31242
2.85	3.010402
2.91	2.403844
2.97	1.832065
3.03	1.389779
3.09	1.113971
3.15	0.938441
3.21	0.836571
3.27	0.775333
3.33	0.751197
3.39	0.749822
3.45	0.76621
3.51	0.781778
3.57	0.800606
3.63	0.8371
3.69	0.861859
3.75	0.894818
3.81	0.924537
3.87	0.957264
3.93	0.979614
3.99	1.008705
4.05	1.03296
4.11	1.068492
4.17	1.090377
4.23	1.096039
4.29	1.111942
4.35	1.124727
4.41	1.123651
4.47	1.129193
4.53	1.129727
4.59	1.124567
4.65	1.113144
4.71	1.109312
4.77	1.096857
4.83	1.076425
4.89	1.062207
4.95	1.040872
5.01	1.017467
5.07	0.998127
5.13	0.986288
5.19	0.958274
5.25	0.944788

5.31	0.922271
5.37	0.91755
5.43	0.905862
5.49	0.89922
5.55	0.893861
5.61	0.884586
5.67	0.887256
5.73	0.889874
5.79	0.901983
5.85	0.910106
5.91	0.922624
5.97	0.935932
6.03	0.95407
6.09	0.966677
6.15	0.985257
6.21	0.998282
6.27	1.011494
6.33	1.024249
6.39	1.037834
6.45	1.044771
6.51	1.056559
6.57	1.058744
6.63	1.064728
6.69	1.065552
6.75	1.066482
6.81	1.067359
6.87	1.06341
6.93	1.05845
6.99	1.053697
7.05	1.051666
7.11	1.044471
7.17	1.038047
7.23	1.031152
7.29	1.019849
7.35	1.014858
7.41	1.009468
7.47	1.002468
7.53	0.995561
7.59	0.992708
7.65	0.988552
7.71	0.986482
7.77	0.982608
7.83	0.980849
7.89	0.978408
7.95	0.981368
8.01	0.981193
8.07	0.980264

8.13	0.985891
8.19	0.987607
8.25	0.990856
8.31	0.991314
8.37	0.99653
8.43	0.996978
8.49	0.999008
8.55	1.001787
8.61	1.007272
8.67	1.006793
8.73	1.005745
8.79	1.010701
8.85	1.010407
8.91	1.013189
8.97	1.009709
9.03	1.009314
9.09	1.011939
9.15	1.008386
9.21	1.005641
9.27	1.008576
9.33	1.004115
9.39	1.007652
9.45	1.004592
9.51	1.003413
9.57	1.000769
9.63	1.001614
9.69	1.003396
9.75	1.000256
9.81	1.001797
9.87	1.001469
9.93	1.001516
9.99	1.00137

<u>Radial distribution function: Oxygen-Hydrogen</u>

**r (A)**	**g(r)**
0.03	0
0.09	0
0.15	0
0.21	0
0.27	0
0.33	0
0.39	0
0.45	0
0.51	0
0.57	0
0.63	0
0.69	0
0.75	0

0.81	0
0.87	0.024154
0.93	12.19058
0.99	27.8425
1.05	1.638486
1.11	0.003049
1.17	0
1.23	0
1.29	0
1.35	0
1.41	0
1.47	0.000297
1.53	0.008281
1.59	0.078972
1.65	0.326867
1.71	0.792794
1.77	1.249703
1.83	1.474016
1.89	1.444864
1.95	1.214764
2.01	0.957082
2.07	0.712961
2.13	0.536714
2.19	0.407028
2.25	0.31955
2.31	0.257481
2.37	0.227024
2.43	2.09E-01
2.49	0.210263
2.55	0.226604
2.61	0.257503
2.67	0.317621
2.73	0.402054
2.79	0.527832
2.85	0.678362
2.91	0.855962
2.97	1.044709
3.03	1.2301
3.09	1.385565
3.15	1.503607
3.21	1.568543
3.27	1.573945
3.33	1.529143
3.39	1.451778
3.45	1.355122
3.51	1.258863
3.57	1.176541

3.63	1.118082
3.69	1.063476
3.75	1.031747
3.81	1.014438
3.87	0.996309
3.93	0.994643
3.99	0.9906
4.05	0.992749
4.11	0.992885
4.17	0.995401
4.23	0.99716
4.29	0.999502
4.35	1.001883
4.41	1.00251
4.47	1.000462
4.53	0.9961
4.59	0.997715
4.65	0.989232
4.71	0.986817
4.77	0.985984
4.83	0.986046
4.89	0.984978
4.95	0.985549
5.01	0.982207
5.07	0.985756
5.13	0.984868
5.19	0.987501
5.25	0.989256
5.31	0.989304
5.37	0.989494
5.43	0.988619
5.49	0.989109
5.55	0.986974
5.61	0.984262
5.67	0.982722
5.73	0.981292
5.79	0.976899
5.85	0.975689
5.91	0.974746
5.97	0.971301
6.03	0.973697
6.09	0.972673
6.15	0.973741
6.21	0.974904
6.27	0.977673
6.33	0.981638
6.39	0.983427

6.45	0.989562
6.51	0.993355
6.57	0.995257
6.63	1.000591
6.69	1.006859
6.75	1.010812
6.81	1.012113
6.87	1.015454
6.93	1.018257
6.99	1.018619
7.05	1.022343
7.11	1.022952
7.17	1.022347
7.23	1.020755
7.29	1.020775
7.35	1.018968
7.41	1.017906
7.47	1.017812
7.53	1.017268
7.59	1.016252
7.65	1.011382
7.71	1.008615
7.77	1.005916
7.83	1.004048
7.89	1.001096
7.95	0.999111
8.01	0.996495
8.07	0.994749
8.13	0.9932
8.19	0.992395
8.25	0.990553
8.31	0.989834
8.37	0.990298
8.43	0.988072
8.49	0.989789
8.55	0.988403
8.61	0.989891
8.67	0.991409
8.73	0.990964
8.79	0.991436
8.85	0.99225
8.91	0.995562
8.97	0.996098
9.03	0.99773
9.09	0.998368
9.15	0.998169
9.21	0.999173

9.27	1.000953
9.33	1.002315
9.39	1.001961
9.45	1.001712
9.51	1.001653
9.57	1.001788
9.63	1.00318
9.69	1.002343
9.75	1.00271
9.81	1.002239
9.87	1.001124
9.93	1.001595
9.99	1.001301

Radial distribution function: Hydrogen-Hydrogen

r (A)	g(r)
0.03	0
0.09	0
0.15	0
0.21	0
0.27	0
0.33	0
0.39	0
0.45	0
0.51	0
0.57	0
0.63	0
0.69	0
0.75	0
0.81	0
0.87	0
0.93	0
0.99	0
1.05	0
1.11	0
1.17	0
1.23	0
1.29	5.64E-05
1.35	0.006729
1.41	0.167947
1.47	1.254815
1.53	3.174416
1.59	2.732198
1.65	0.826407
1.71	0.105426
1.77	0.05707

1.83	0.123514
1.89	0.239343
1.95	0.410865
2.01	0.643587
2.07	0.892752
2.13	1.134793
2.19	1.308311
2.25	1.410388
2.31	1.421971
2.37	1.362253
2.43	1.25E+00
2.49	1.116658
2.55	0.984839
2.61	0.873665
2.67	0.778963
2.73	0.714479
2.79	0.675067
2.85	0.661228
2.91	0.662358
2.97	0.678404
3.03	0.708772
3.09	0.750753
3.15	0.795414
3.21	0.845761
3.27	0.897028
3.33	0.952814
3.39	1.006647
3.45	1.062963
3.51	1.112094
3.57	1.160918
3.63	1.193126
3.69	1.216295
3.75	1.22449
3.81	1.212814
3.87	1.195951
3.93	1.169713
3.99	1.141791
4.05	1.112755
4.11	1.084195
4.17	1.064942
4.23	1.047492
4.29	1.035526
4.35	1.0232
4.41	1.017948
4.47	1.013815
4.53	1.009237
4.59	1.000754

4.65	1.000526
4.71	0.995271
4.77	0.995168
4.83	0.989775
4.89	0.985605
4.95	0.983347
5.01	0.97933
5.07	0.978248
5.13	0.972536
5.19	0.970972
5.25	0.973592
5.31	0.968774
5.37	0.970262
5.43	0.968444
5.49	0.973319
5.55	0.977351
5.61	0.978119
5.67	0.980124
5.73	0.986306
5.79	0.990143
5.85	0.991831
5.91	0.993338
5.97	0.994396
6.03	0.995282
6.09	0.999084
6.15	0.997317
6.21	0.998058
6.27	0.99954
6.33	0.99907
6.39	0.998828
6.45	0.998722
6.51	1.000555
6.57	0.999491
6.63	1.001048
6.69	0.999765
6.75	1.002347
6.81	1.003822
6.87	1.004365
6.93	1.005984
6.99	1.006687
7.05	1.008156
7.11	1.010415
7.17	1.009363
7.23	1.009695
7.29	1.010871
7.35	1.012091
7.41	1.012847

7.47	1.010569
7.53	1.010783
7.59	1.010717
7.65	1.010239
7.71	1.009973
7.77	1.010331
7.83	1.009145
7.89	1.007804
7.95	1.005945
8.01	1.006637
8.07	1.003857
8.13	1.002889
8.19	1.003499
8.25	1.000895
8.31	1.001943
8.37	1.000482
8.43	0.999804
8.49	0.999698
8.55	0.99736
8.61	0.99815
8.67	0.998451
8.73	0.996769
8.79	0.997845
8.85	0.996562
8.91	0.996836
8.97	0.997641
9.03	0.996381
9.09	0.997686
9.15	0.998126
9.21	0.997849
9.27	0.999527
9.33	0.999615
9.39	0.999805
9.45	1.000636
9.51	1.0024
9.57	1.002826
9.63	1.004419
9.69	1.002805
9.75	1.003466
9.81	1.003775
9.87	1.003276
9.93	1.00383
9.99	1.00281

References

1. Popelier, P. L. A.; Joubert, L.; Kosov, D. S., Convergence of the Electrostatic Interaction Based on Topological Atoms. *J.Phys.Chem.A* **2001,** *105*, 8254-8261.
2. Kosov, D. S.; Popelier, P. L. A., Convergence of the multipole expansion for electrostatic potentials of finite topological atoms. *J.Chem.Phys.* **2000,** *113*, 3969-3974.
3. Popelier, P. L. A.; Rafat, M., The electrostatic potential generated by topological atoms: a continuous multipole method leading to larger convergence regions. *Chem.Phys.Lett.* **2003,** *376*, 148-153.
4. Rafat, M.; Popelier, P. L. A., A convergent multipole expansion for 1,3 and 1,4 Coulomb interactions. *J.Chem.Phys.* **2006,** *124*, 144102-1-7.
